# The postcranial anatomy of *Brasilodon quadrangularis* and the acquisition of mammaliaform traits among non-mammaliaform cynodonts

**DOI:** 10.1371/journal.pone.0216672

**Published:** 2019-05-10

**Authors:** Morgan L. Guignard, Agustin G. Martinelli, Marina B. Soares

**Affiliations:** 1 Programa de Pós-Graduação em Geociências, Universidade Federal do Rio Grande do Sul, Agronomia, Porto Alegre, Rio Grande do Sul, Brazil; 2 CONICET-Sección Paleontología de Vertebrados, Museo Argentino de Ciencias Naturales ’Bernardino Rivadavia', Buenos Aires, Argentina; 3 Departamento de Paleontologia e Estratigrafia, Instituto de Geociências, Universidade Federal do Rio Grande do Sul, Agronomia, Porto Alegre, Rio Grande do Sul, Brazil; Universiteit Maastricht, NETHERLANDS

## Abstract

*Brasilodon quadrangularis* (Cynodontia, Probainognathia) is an iconic non-mammaliaform cynodont from the Late Triassic of Brazil (*Riograndia* Assemblage Zone, Candelária Sequence), being considered as the sister taxon of Mammaliaformes. Although its phylogenetic position is very important, several aspects of its postcranial anatomy remain unclear or unstudied. Here, we present a detailed description of the postcranial elements referred to *Brasilodon*, including previously mentioned specimens and new ones, which add relevant information about its postcranial morphology and provide a new insight into the anatomical transition between advanced non-mammaliaform cynodonts and early mammaliaforms. Functional and ecological implications are also investigated, based on the postcranial morphology and muscular reconstructions. The postcranium of *Brasilodon* differs from most non-mammaliaform cynodonts and presents similarities with tritylodontids, early mammaliaforms and extant therians, such as a ventrally oriented scapular glenoid facet, a distinct and ossified greater humeral tubercle, lack of ectepicondylar foramen, olecranon process, hemispherical humeral and femoral heads and a prominent intertrochanteric crest. The humeral torsion, the length of the deltopectoral crest, the large bicipital groove and the well-developed lesser tubercle, indicate that the forelimb of *Brasilodon* was hold in a semi-sprawling position, with well-developed adductor muscles to maintain the body off the ground. The short femoral neck and the strong medial projection of the femoral head indicate the femur was held in a more erect posture than in basal non-mammaliaform cynodonts. The anterodorsally projected iliac blade with reduced postacetabular process, reduction of the anterior part of the pubis, medially located lesser trochanter indicate a basically mammalian pattern of pelvic musculature, able to swing the femur in a nearly parasagittal plane.

## Introduction

Much attention has been paid to the craniomandibular changes in the non-mammaliaform cynodont to mammaliaform transition (e.g., [1–8]). Comparatively, the postcranial skeleton of derived non-mammaliaform probainognathians is less known, considering that non-mammaliaform cynodont systematic is mainly based upon dental and cranial features (e.g., [7, 9–10]). Moreover, a general view is that the postcranial skeleton of non-mammaliaform cynodonts is quite conservative compared to craniodental adaptations, having an “intermediate bauplan” between basal therapsids and early mammaliaforms [11]. Nonetheless, the craniodental disparity among Triassic cynodonts also reflects postcranial diversity, with small to large body sizes, possibly aquatic, or semi-fossorial to fossorial forms (e.g., [11–21]). In addition, the postcranial anatomy in Triassic cynodonts permits to evaluate the main changes and locomotor advances in mammalian evolution and provide information about the transition from the sprawling posture of basal synapsids to a more erect posture. Probainognathian cynodonts, the clade which include the crown Mammalia (e.g., [22–23]), have an extremely conspicuous fossil record in South America, represented by chiniquodontids (e.g., *Chiniquodon theotonicus*, *Aleodon cromptoni*), ecteniniids (e.g., *Ecteninion lunensis*, *Trucidocynodon riograndensis*), probainognathids (*Probainognathus jenseni*, *Bonacynodon schultzi*), ictidosaurians (e.g., *Riograndia guaibensis* plus tritheledontids) and other prozostrodontians (e.g., *Prozostrodon brasiliensis*, *Therioherpeton cargnini*, *Brasilodon quadrangularis*). Although the relevant phylogenetic position of *Brasilodon quadrangularis* as sister taxon of Mammaliaformes (e.g., [5, 7, 24–26]), many aspects of its postcranial anatomy remain unclear. Some postcranial elements were briefly described and/or mentioned by Bonaparte et al. [5, 27], but in-depth studies were never developed. Here, we present a detailed description of the postcranial elements of *Brasilodon quadrangularis*, including previously mentioned specimens and new ones. It adds more information about its postcranial morphology and provides new insights into the anatomical transition between the advanced non-mammaliaform cynodonts and early mammaliaforms. Moreover, we also investigate functional implications of its postcranium to improve our knowledge on posture and locomotion of mammalian precursors.

## Materials and methods

### Access to specimens

The specimens described here and those used for comparison belong to public collections and were examined with the explicit permission of appropriate curators and/or collection managers (see Acknowledgments). Repository locations and abbreviations for all specimens cited in the text are listed below. We followed all Brazilian regulations for fossil studies and we complied with the PLoS Paleontological Ethics Statement. In Table 1, we listed selected cynodont species and specimens here used for comparisons.

**Table 1:** List of specimens used for comparisons.

Taxa	Specimens
**Non-mammaliaform cynodonts**	
*Aleodon cromptoni*	UFRGS-PV-0146-T
*Brasilodon quadrangularis*	UFRGS-PV-1043-T
*Cricodon metabolus*	UMZC T905
*Cynognathus crateronotus*	NHMUK-R2571
*Cynognathus* sp.	NHMUK-R3772a
*Diademodon* sp.	NHMUK-R2803, R3581; UMZC T433, T436, T447-449, T455-456, T489-502, T503, T971, T1017
*Exaeretodon* sp.	MACN-PV s/n (col. 1958)
*Galesaurus planiceps*	UMZC T823
*Galesaurus* sp.	UMZC T820-821
*Irajatherium hernandezi*	UFRGS-PV-599-T, PV-1068-T
*Luangwa drysdalli*	OUMNH-TSK121
*Oligokyphus* sp.	NHMUK-R7386-7491
*Pascualgnathus polanskii*	MLP-65-V1-18-1
*Prozostrodon brasiliensis*	UFRGS-PV-0248-T
*Scalenodon angustifrons*	UMZC T925, T972
*Scalenodon* sp.	NHMUK-R36802, R9391; UMZC T974
*Therioherpeton cargnini*	MVP-05.22.04
*Thrinaxodon liorhinus*	NHMUK-R15957; UMZC T1101
*Trucidocynodon riograndensis*	UFRGS-PV-1051-T
**Mammaliaforms**	
*Megazostrodon rudnerae*	NHNUK M26407
*Vincelestes neuquenianus*	MACN N01, N09, N37-N39
*Ornithorhynchus anatinus*	MNHN 1906–484
*Tachyglossus aculeatus*	MNHN 1903–537, 1903–538
*Caluromys philander*	MNHN 1999–1061
*Didelphis marsupialis*	MNHN 1978–538
*Marmosa demerarae*	MNHN 1998–1832
*Canis lupus familiaris*	UFRGS-PV-14-Z

### Materials

The following description is mainly based on twospecimens housed at the Departamento de Paleontologia e Estratigrafia, Instituto de Geociências, Universidade Federal do Rio Grande do Sul (UFRGS), Porto Alegre, Brazil. The specimen UFRGS-PV-0765-T includes an incomplete skull, partial right and left lower jaws, a right ulna, phalanges and some fragments of metapods and indeterminate bones. The ulna was figured and briefly commented by Bonaparte et al. [5], and originally referred as to *Brasilodon quadrangularis*. The specimen UFRGS-PV-1043-T consists of an almost complete skull, lower jaws and an incomplete and disarticulated axial and appendicular skeleton, including incomplete vertebrae and ribs, right scapula, left humerus, left radius, ulnae, left acetabulum, right femur, left tibia, left calcaneum, left astragalus, fragments of metapods and phalanges. It was originally described as *Brasilitherium riograndensis* [27]. Some of its appendicular bones (humerus, ulna, radius, femur and tibia) were figured but briefly described or not at all [27]. The skull of UFRGS-PV-1043-T was also used for the reconstruction of the inner ear, brain and nasal cavities [28–30]. Most postcranial elements are well-preserved, including the epiphysis in long bones. Both UFRGS-PV-0765T and UFRGS-PV-1043T were used in the histological study by Botha-Brink et al. [31]. Here, *Brasilitherium riograndensis* is considered as the junior synonym of *Brasilodon quadrangularis*, following Liu and Olsen, [7]. This hypothesis is also supported by the histological study (see [31]).

These postcranial elements were compared to some non-mammaliaform cynodonts, Mesozoic mammal lineages (e.g., *Vincelestes neuquenianus*) and extant mammals (monotremes, marsupials and placentals) listed in Table 1. Moreover, comparisons with other non-mammaliaform cynodonts (e.g., *Procynosuchus*, *Andescynodon*, *Tritylodon*), early mammaliaforms (e.g., *Morganucodon* (“*Eozostrodon*”), *Haldanodon*) and Mesozoic mammal lineages (e.g., *Gobiconodon*, *Catopsbaatar*, *Akidolestes*, *Zhangheotherium*) were also made on the basis of detailed descriptions in the literature (e.g., [14, 19, 32–40]).

The postcranial measurements were taken directly on the specimens with a digital caliper to the nearest 0.1 mm. Angle measurements were made with the software Inkscape, based on pictures of the material (Table 2). Some structures (fossae, crests) present on the appendicular skeleton of *B*. *quadrangularis* permit the determination of the approximate position of muscles. The origin and insertion of main muscles were estimated using homology models including non-mammaliaform therapsids ([13, 41–43]) and studies dealing with osteology and myology of living amphibians ([44–46]), squamates ([47–49]), crocodylians ([50–53]), monotremes ([54–57]), metatherians ([58–61]), and eutherians ([62–67]).

**Table 2:** Angles and indices of non-mammaliaform cynodonts. **TH**, torsion of humeral shaft; **ADC**, angle of deltopectoral crest; **LDC**, length ratio of deltopectoral crest (Length of deltopectoral crest / Humeral length * 100); **DW**, width ratio of distal end (Distal end width / Humeral length). The angle TH was measured between the transversal axes of the proximal end (the axis “Lesser Tuberosity—Medial margin of the humeral head”) and the distal end (the axis “Ectepicondyle-Entepicondyle”), based on the photography of the humerus in proximal view. The angle ADC was measured between the deltopectoral crest and the transversal axis of the proximal end, based on the photography of the humerus in proximal view. The distal end width was measured between the two epicondyles, based on the photography of the humerus in anterior view.

Taxa	TH	ADC	LDC	DW	References
*Procynosuchus*	-	145°	52%	49%	Abdala, 1999
*Galesaurus*	20–40°	132°-135°	50%	50%	UMZC-T820/T823
*Thrinaxodon*	40–50°	135°	50%	50%	Jenkins, 1971
*Platycraniellus*	-	90°	-	-	Abdala, 2007
*Cynognathus*	20°	125°	60%	51%	NHMUK-R3772a
*Diademodon*	20°	105–120°	58%	44%	UMZC-T492/T433
*Cricodon*	40°	-	50%	42%	Crompton, 1954
*Scalenodon*	5°	100°	50%	38%	UMZC-T925
*Massetognathus*	-	90°	52%	38–40%	Jenkins, 1971; Abdala, 1999
*Luangwa*	5°	102°	53%	40%	OUMNH-TSK121
*Andescynodon*	-	90°	54–61%	-	Abdala, 1999
*Boreogomphodon*	-	105°	50%	60%	Liu et al., 2017
*Exaeretodon*	5°-30°*	150°	54%	56%	*Bonaparte, 1963
*Pascualgnathus*	15°	95°	52%	45%	MLP-65-V1-18-1
*Santacruzodon*	65°	-	-	-	Bertoni, 2014
*Trucidocynodon*	-	90°	50%	30%	UFRGS-PV-1051-T
*Chiniquodon*	-	125°	54–57%	58–63%	Abdala, 1999
*Probainognathus*	-	135°	45–50%	34–37%	Abdala, 1999
*Prozostrodon*	5°	95°	45%	58%	UFRGS-PV-0248-T
*Irajatherium*	-	90°	53%	55%	UFRGS-PV-599-T
*Riograndia*	22°	100°	50%	54%	UFRGS-PV-833-T
*Bienotheroides*	20–45°	-	-	-	Sun et al., 1985
*Tritylodon*	30–40°	100°	50%	48–51%	Gaetano et al., 2017
*Oligokyphus*	40°	95°	52%	42%	NHMUK-R7402; Kühne, 1956
*Kayentatherium*	40°	-	53%	60%	Sues and Jenkins, 2006
*Brasilodon*	15°	100°	48%	43%	UFRGS-PV-1043-T
*Morgancudon*	50°	-	44%	30%	Jenkins and Parrington, 1976
*Haldanodon*	60°	-	-	61%	Martin, 2005
*Gobiconodon*	33°	-	-	43%	Jenkins and Schaff, 1988
*Akidolestes*	40°	-	-	-	Chen and Luo, 2012
*Zhangheotherium*	30°	-	-	-	Hu et al., 1997
*Vincelestes*	25°	120°	44%	38%	MACN-N09
*Tachyglossus*	60°	95°	48%	95%	MNHN-1903-537
*Ornithorhynchus*	75°	105°	54%	83%	MNHN-1906-484
*Didelphis*	<5°	-	55%	30%	MNHN-1878-538
*Canis*	<5°	-	41%	20%	UFRGS-PV-14-Z

“Mammalian” rather than “reptilian” terminology and orientation are used to designate bone structures and muscles, but the “reptilian” homologue of each structure and muscle is given the first time the structure/muscle is mentioned. Muscle nomenclature and homology between mammals and reptiles are mainly based on the work of Ellsworth ([68]), Diogo et al. ([69]), and Abdala and Diogo ([70]).

### Institutional abbreviations

MACN, Museo Argentino de Ciencias Naturales “Bernardino Rivadavia”, Buenos Aires, Argentina; MLP, Museo de La Plata, La Plata, Argentina; MNHN, Museum National d’Histoire Naturelle, Paris, France; MVP, Museu do Patronato Alves Ramos, Santa Maria, Brazil; NHMUK, Natural History Museum, London, United Kingdom; OUMNH, Oxford University Museum of Natural History, Oxford, United Kingdom; UFRGS, Universidade Federal do Rio Grande do Sul, Porto Alegre, Brazil; UMZC, University Museum of Zoology, Cambridge, United Kingdom.

## Results

### Systematic paleontology

CYNODONTIA Owen, 1861

PROBAINOGNATHIA Hopson, 1990

PROZOSTRODONTIA Liu and Olsen, 2010

BRASILODONTIDAE Bonaparte, Martinelli and Schultz, 2005

*BRASILODON* Bonaparte, Martinelli, Schultz and Rubert, 2003

*BRASILODON QUADRANGULARIS* Bonaparte, Martinelli, Schultz and Rubert, 2003

#### Holotype

UFRGS-PV-0611-T, incomplete skull with complete upper postcanine teeth.

#### Referred material

Many specimens of this species are known, originally referred to *B*. *quadrangularis* ([5, 24, 71–72]), *Brasilitherium riograndensis* ([5, 24, 27, 71–72]), and *Minicynodon maieri* ([27, 71]). Two latter species are considered junior synonym of *B*. *quadrangularis* (see [7, 73]). A detailed list of main specimens can be found in Martinelli [73].

#### Emended diagnosis

Small-sized probainognathian cynodont (~20 to ~40 mm of basal skull length) with the following association of features (autapomophies marked with an asterisk): upper postcanine teeth with a central, large cusp (A) and a mesial (B) and a distal (C) cusp, symmetrically arranged on the lingual side of the crown, with small accessory mesiolabial and distolingual cusps; “triconodont-like” lower postcanine teeth with lingual accessory cusps; lower middle postcanine teeth with mesiolingual cusp e and lingual cusp g; presence of a tongue (by cusp d) and groove-like (by cusps b/e) mesiodistal interlocking system among middle lower postcanine teeth; upper/lower diastema between canines and postcanines in adult specimens; lower first incisor small and procumbent; postcanines with constricted root, with two separated nutritious canals; slender zygomatic arch; absence of postorbital bar; absence of prefrontal and postorbital bones; fused ophistotic and prootic (i.e., petrosal) with well-defined promontorium; long secondary palate; presence of interpterygoid vacuities; separate foramina for nerves V2 and V3; quadrate with a well-developed stapedial process; unfused lower jaw; symphysis anterodorsally projected; articular process of dentary reaching a more posterior position than the surangular, possibly reaching squamosal (but without a condyle); prominent tuberosity for *M*. *teres major*; expanded distal end representing about 43% of humeral length; lack of ectepicondylar foramen in humerus; short femoral neck; salient intertrochanteric crest (modified from Bonaparte et al. [5]; Martinelli [73]).

#### Locality and horizon

**Fig 1:**
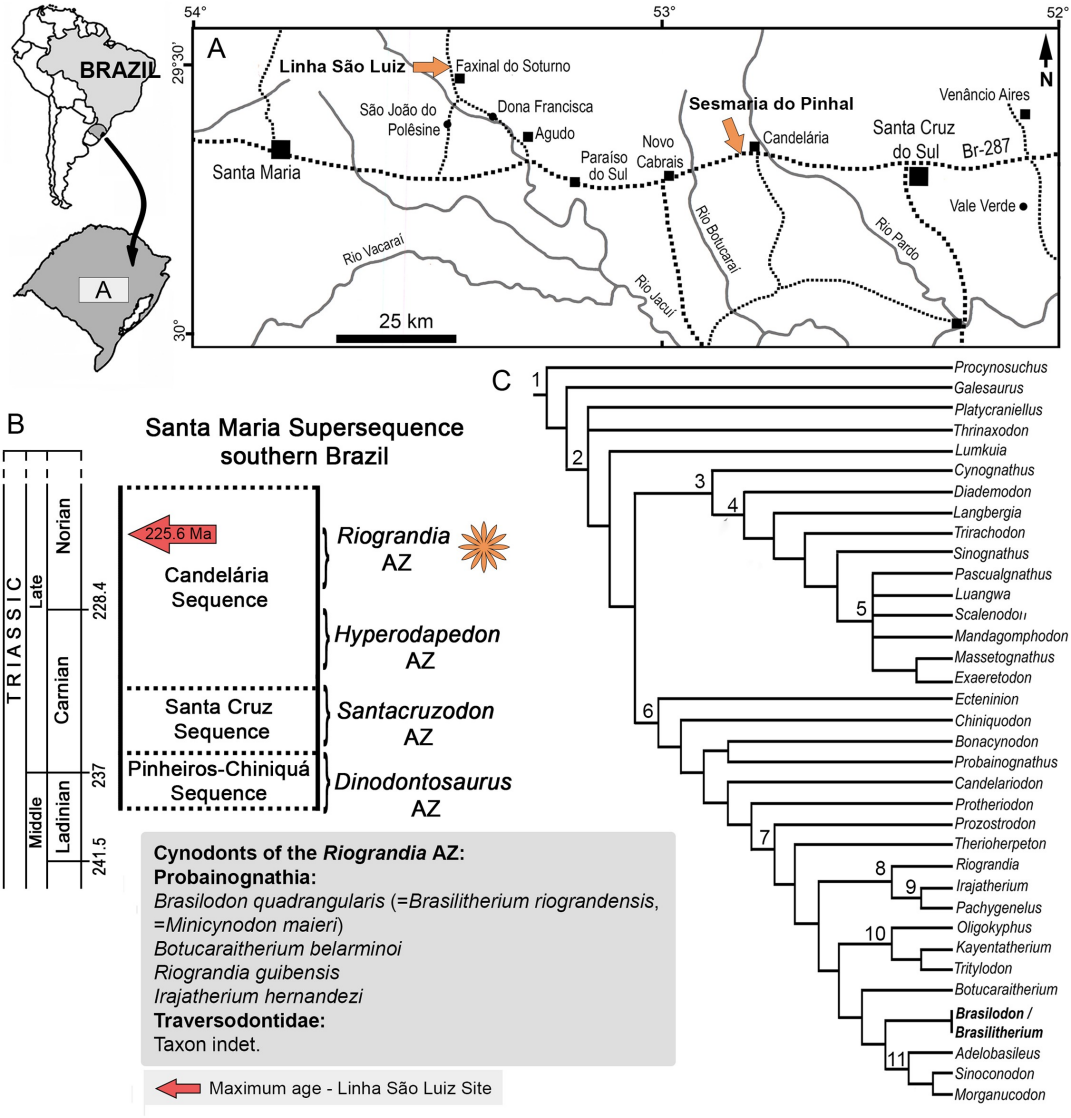
Localities and stratigraphic level with *Brasilodon quadrangularis*. **A**, location map showing the Linha São Luis and Sesmaria do Pinhal sites, in Rio Grande do Sul state, southern Brazil; **B**, chrono- and biostratigraphy of Triassic units with vertebrate assemblage zones (AZ); **C**, compositive phylogenetic position of *Brasilodon* amongst Probainognathia (based on [6–7, 25]). Clade names: 1, Cynodontia; 2, Epicynodontia; 3, Cynognathia; 4, Gomphodontia; 5, Traversodontidae; 6, Probainognathia; 7, Prozostrodontia; 8, Ictidosauria; 9, Tritheledontidae; 10, Tritylodontidae: 11, Mammaliaformes. The ages (Ma) of the column follow Ogg et al. [78], chrono- and biostratigraphy were modified from Zerfass et al. [75] and Horn et al. [76], the age of *Riograndia* AZ was taken from Langer et al. [77].

Specimens referred to *B*. *quadrangularis* were found on the Linha São Luiz and the Sesmaria do Pinhal sites, from the municipalities of Faxinal do Soturno and Candelária, respectively, Rio Grande do Sul state, Brazil (Fig 1). The stratigraphic level corresponds to the *Riograndia* AZ [74] of the Candelária Sequence, Santa Maria Supersequence [75–76], which is considered Norian, based on a recent dating of maximum deposition age of 225.42 ± 0.37Ma [77].

### Description and comparisons

#### Axial skeleton

**Fig 2:**
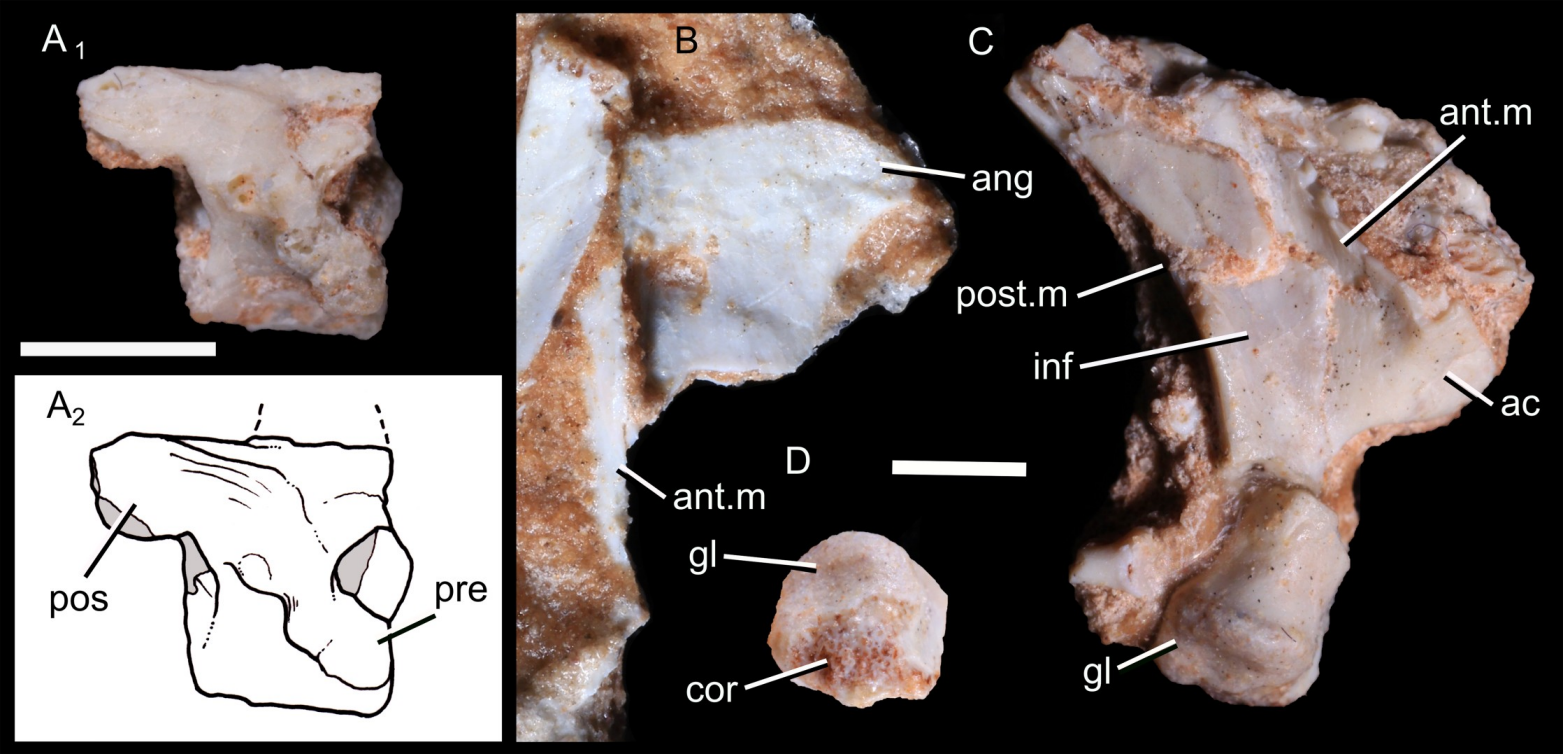
Vertebra and scapula of *Brasilodon quadrangularis* (UFRGS-PV-1043-T). **A**, photography (1) and drawing (2) of a fragmented vertebra; **B**, photography of the possible dorsal part of the right scapula, in medial view; **C**, photography of the ventral part of the right scapula, in lateral view; **D**, photography of the glenoid, in distal view. **Abbreviations: ac**, acromion; **ang**, posterodorsal angle; **ant.m**, anterior scapular margin; **cor**, area of contact with the coracoid; **gl**, scapular glenoid facet; **inf**, infraspinous (supracoracoideus) fossa; **pos**, postzygapophysis; **post.m**, posterior scapular margin; **pre**, prezygapophysis. Scale bar = 2 mm.

**Vertebrae:** The specimen UFRGS-1043-T includes four isolated and poorly preserved presacral vertebrae, bearing the centra with eroded neural arches (Fig 2A). The centra are flat on the anterior side and concave on the posterior side (platycoelous), as seen in some tritylodontids such as *Tritylodon* and *Bienotheroides* [19] and in the early mammaliaform *Morganucodon* [32]. Centra are deeply amphicoelous in most non-mammaliaform cynodonts (e.g., [12, 17–18, 38, 79–80]). The neural arches are poorly preserved, almost all vertebral processes are broken. The neural arch is fused with the centrum with no evidence of neurocentral suture, indicating the adult nature of the specimen (one of the largest known *Brasilodon* specimen; see also [31]). The neural canal is relatively large, with thin lateral walls. The base of the diapophysis is present on one element, it is small and circular in cross-section. No accessory zygapophyseal articulations (anapophyses) can be observed. Anapophyses occur in all basal epicynodonts (e.g., *Galesaurus*, *Thrinaxodon*) and several cynognathians (e.g., *Cynognathus*, *Diademodon*, *Cricodon*, *Andescynodon*, *Luangwa*, *Protuberum*; [17, 38]). They are not observed in the axial skeleton of *Procynosuchus* [14], several traversodontids (e.g., *Menadon*, *Massetognathus*, *Exaeretodon*; [12, 81–82] and probainognathians [16, 80, 83]. The pre- and postzygapophyses are eroded but postzygapophyses appear to have a nearly horizontal orientation and are placed in a relatively high position on the neural arch (Fig 2A). The base of the neural spine suggests it is anteroposteriorly long and laminar, but its real high is unknown.

**Ribs:** The specimen UFRGS-1043-T includes four isolated fragments of presacral ribs. The shaft is curved and anteroposteriorly flattened. All preserved ribs lack the expanded costal plates or processes. Costal plates are represented in all basal epicynodonts in which the postcranium is known [13, 84] and most cynognathians (e.g., *Cynognathus*, *Diademodon*, *Luangwa*, *Andescynodon*, *Menadon*, *Protuberum*, *Pascualgnathus*, and *Scalenodon*; [17, 38, 80, 82]). They were lost in several traversodontids (e.g., *Exaeretodon*, *Boreogomphodon*; [80, 85]) and all probainognathians (e.g., [18, 43, 83]).

#### Pectoral girdle and forelimb

**Scapula:** The specimen UFRGS-PV1043T includes the ventral part of the right scapula. An associated and damaged bone could be the medial surface of the dorsal part of the right scapula and a flange could be its anterior border (Fig 2B). The dorsal margin is slightly convex and the posterodorsal angle is posteriorly projected. A pointed posterodorsal angle is also observed in *Trucidocynodon*, but not in other non-mammaliaform cynodonts.

Although it is incomplete, the scapular blade appears to be thin and narrow (Fig 2C). An anteroposterior constriction occurs on the base, above the glenoid, as seen in other epicynodonts (e.g., *Thrinaxodon*, *Galesaurus*, *Cynognathus*, *Luangwa*, *Trucidocynodon*, *Riograndia*). The posterior margin of the blade consists of a laterally projected flange. The posterior surface of this flange is damaged, so it is unsure if a postscapular fossa, as described for tritylodontids and *Riograndia*, is present [16, 19, 21]. The flange on the anterior margin is also laterally projected. Ventrally, this flange is everted and faces anteriorly. Laterally projected anterior and posterior margins are typical in the scapulae of non-mammaliaform epicynodonts. The anterior margin ends in a well-developed acromion. It consists of a tuberosity protruding anteriorly, as in *Cynognathus*, *Diademodon*, *Luangwa*, *Menadon* [82], *Boreogomphodon* [85], *Exaeretodon* [86], *Trucidocynodon*, *Probainognathus* [87], *Chiniquodon* [87], *Pachygenelus* [88] and tritylodontids [16, 19, 89]. According to Jenkins [13], there is no acromion process in *Thrinaxodon* but only a local thickening on the ventral part of the anterior edge. Nothing is preserved anteriorly to the anterior flange, and a supraspinous fossa was probably absent in *Brasilodon*, as in most non-mammaliaform cynodonts. A deep infraspinous (supracoracoideus) fossa is present on the lateral surface of the blade, enclosed by the anterior and posterior borders.

The glenoid is preserved but separated from the blade (Fig 2C and 2D). The scapular glenoid facet is concave and hemi-ovoidal. It faces ventrally as in *Riograndia*, *Kayentatherium* [13], *Tritylodon* [19], rather than posteroventrally as in *Procynosuchus* [14], *Thrinaxodon* [13], *Galesaurus* [84], *Cynognathus*, *Menadon* [82] and *Luangwa*. The medial margin of the glenoid is straight, representing the area of contact with the coracoid. Anterodorsal to the glenoid facet, the base of the scapula extends slightly anteriorly to form a flange below the acromion. This flange is broken and should be the articular surface for the procoracoid.

**Humerus:** The following description is based on the specimen UFRGS-PV1043T, which includes a complete and well-preserved left humerus, available in all views. The bone is described with a mammalian orientation, with the shaft held vertically and the deltopectoral crest facing anteriorly.

**Fig 3:**
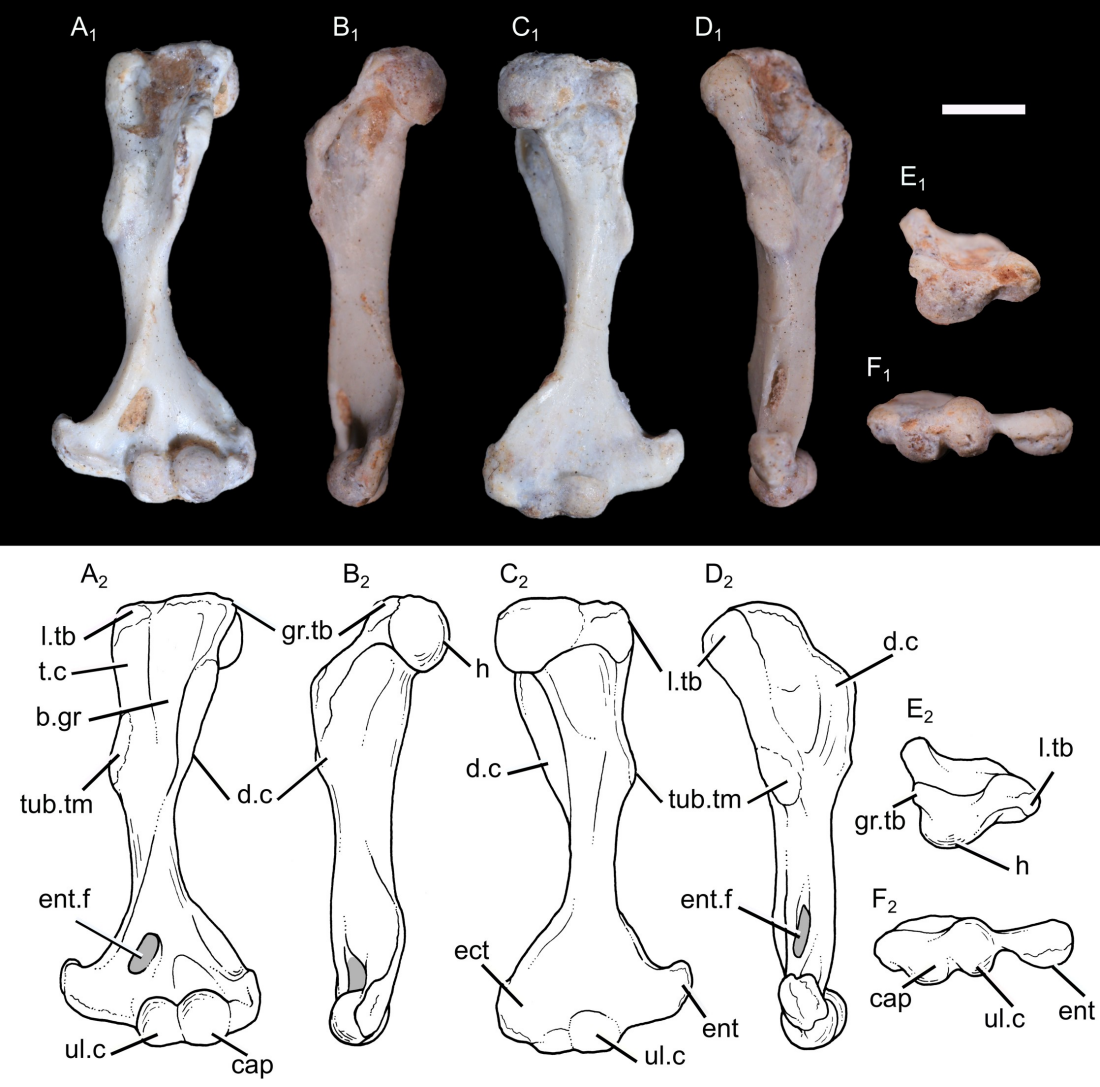
Photography (1) and drawing (2) of the left humerus of *Brasilodon quadrangularis* (UFRGS-PV-1043-T). **A**, in anterior view; **B**, in lateral view; **C**, in posterior view; **D**, in medial view; **E**, in proximal view; **F**, in distal view. **Abbreviations: b.gr**, bicipital groove; **cap**, capitulum; **d.c**, deltopectoral crest; **ect**, ectepicondyle; **ent**, entepicondyle; **ent.f**, entepicondylar foramen; **gr.tb**, greater tubercle; **h**, humeral head; **int.gr**, intercondylar groove; **l.tb**, lesser tubercle; **t.c**, teres crest; **tub.tm**, tuberosity for the origin of the *M*. *teres major*; **ul.c**, ulnar condyle. Scale bar = 3 mm.

The humerus is 15.6 mm long, with a slender shaft (Fig 3). It is slightly twisted with the transversal axis of the proximal and distal ends forming an angle of 15° in proximal view. A twisted humerus is present in most non-mammaliaform cynodonts, the early mammaliaform *Morganucodon*, the docodont *Haldanodon*, the eutriconodont *Gobiconodon*, the spalacotheriid *Akidolestes* and *Zhangheotherium*, the cladotherian *Vincelestes* and monotremes (Table 2), but the torsion is very small or absent in most therians [90].

The humeral head is perfectly hemispherical, as in mammaliaforms, including extant therians (e.g., [32, 36, 39]). In other non-mammaliaform cynodonts, the articular surface of the head is more ovoid, with the long axis extending mediolaterally, and the dorsoventral curvature is less extensive than that seen in *B*. *quadrangularis*. If the shaft is held with a vertical orientation, the head faces posterolaterally, as in other non-mammaliaform cynodonts, *Morganucodon*, *Haldanodon* and *Vincelestes* (e.g., [15, 18, 27, 91–92]) but differing from the posteriorly facing humeral head of most living therians. The humeral head of *B*. *quadrangularis* extends only on the posterior surface of the humerus, and does not protrude above the proximal outline of the humerus. On the posterior surface, a ridge runs distally from the humeral head and become confluent with the ectepicondylar crest at the distal end of the bone.

The greater tubercle of *B*. *quadrangularis* is a very small but distinct tubercle, located between the head and the proximal margin of the deltopectoral crest. It is confluent with the deltopectoral crest, but separated from the humeral head by a small groove. In tritylodontids and more basal non-mammaliaform cynodonts, the area representing the greater tubercle is confluent with the humeral head (although the head may have been more prominent as part of a cartilaginous epiphyseal cap). In contrast, the humerus of early mammaliaforms has distinct bony tubercles, a pattern retained in more derived mammals [16, 32, 36]. The lesser tubercle is a thick and bulbous bony tuberosity, which protrudes medially and stands apart from the humeral head. The lesser tubercle is larger than the greater tubercle. This feature is a condition observed in other non-mammaliaform cynodonts, early mammaliaforms (e.g., *Morganucodon*, *Haldanodon*) and monotremes, whereas the lesser tubercle is smaller than the greater one in the spalacotheriids and extant therians [34, 36, 39]. The humerus of multituberculates is characterized by a lesser tubercle that is only slightly smaller than the greater tubercle [93] and the tubercles of *Vincelestes* have approximately the same size. On the posterior surface, a shallow and oval fossa is located distally to the lesser tubercle.

The deltopectoral crest is a rectangular flange projected almost perpendicularly to the transversal axis of the proximal end. The crest extends for approximately half the length of the humerus, a common feature in non-mammaliaform cynodonts (Table 2). The crest is thicker along its apex. It is greatly expanded anteriorly in *B*. *quadrangularis* compared to *Vincelestes* and most therians [21, 43]. A robust deltopectoral crest is also present in other non-mammaliaform cynodonts, early mammaliaforms, monotremes and digging therians (e.g., *Talpa*, *Amblysomus*, *Desmana*, *Scalops*; [36, 94]). On the lateral side of the crest, there is a well-defined and deep fossa, becoming shallower distally.

The teres crest originates from the lesser tubercle and extends as far distally as the deltopectoral crest, near the mid-portion of the bone. A similar crest is seen in non-mammaliaform cynodonts, early mammaliaforms and most mesozoic mammals, but not in *Vincelestes* and living therians. A large and oval-shaped osseous process occurs at the distal extremity of the eres crest, probably for the insertion of the *M*. *teres major*. Jenkins [12–13] observed a groove located in the same area in *Thrinaxodon*, *Cynognathus* and the traversodontid *Massetognathus*. A tuberosity is also present in *Exaeretodon*, *Luangwa*, *Pascualgnathus* [95], *Riograndia* [21], *Irajatherium*, *Oligokyphus*, *Morganucodon* [32], *Haldanodon* [36] and *Vincelestes*. Such a tuberosity is absent in multituberculates, spalacotheriids and extant therians [37, 39]. As in other non-mammaliaform cynodonts, early mammaliaforms, multituberculates and *Akidolestes*, the deltopectoral crest and the teres crest enclose a wide and concave area, the bicipital or intertubercular groove, on the anterior surface of the humerus. This broad groove is well-marked proximally but becomes indistinct by the mid-length of the shaft.

Distally, the deltopectoral crest decreases gradually and become a small but well-defined ridge. This ridge is anteriorly projected but is everted distally toward the medial surface of the bone, and connect the bridge of the entepicondylar foramen. The entepicondyle is robust and prominent medially, representing one third of the distal articulation. Distally, it nearly reaches the same level of the capitulum and ulnar condyle. The most medial part of the entepicondyle is a process projecting proximally. The extremity of the entepicondyle represents a thick surface in distal view. The ectepicondylar crest forms a wide surface extending along the distal half of the humeral diaphysis, with a shallow fossa facing anteriorly. The ectepicondyle is narrower and does not protrude as far laterally as the entepicondyle. The distal extremity is wide and the width across the epicondyles is 43% of the total proximodistal length of the bone (Table 2). The distal end is narrower than most non-mammaliaform cynodonts (e.g., *Galesaurus*, *Thrinaxodon*, *Cynognathus*, *Exaeretodon*, *Prozostrodon*, *Boreogomphodon*, *Chiniquodon*, *Riograndia*, *Irajatherium*, *Kayentatherium*), *Haldanodon*, *Gobiconodon* and monotremes, but still wider than the distal end of *Morganucodon* or *Didelphis*.

The entepicondylar foramen is oval and large. It is enclosed by a stout flange of the bone and prolonged by a groove which continues to the distal end. This groove separates the prominent entepicondyle and the distal articular facet. There is no ectepicondylar foramen. The ectepicondylar foramen is present in most non-mammaliaform cynodonts (e.g., [13–15, 38, 40, 84, 96]) but is lacking in tritylodontids [16, 19, 89, 97–99], *Boreogomphodon* [85], *Probainognathus* [79], *Trucidocynodon* [18], early mammaliaforms [32, 36], and most mammals (e.g., [13]).

The distal articular surface is represented by two bulbous condyles. The ulnar condyle is located on the middle of the distal extremity of the bone. Anteriorly, this condyle is transversally compressed. It wraps around the distal end and extends onto both anterior and posterior aspects of the humerus. A bulbous ulnar condyle is also observed in other non-mammaliaform cynodonts [14, 16, 18, 27, 42, 79, 91–92], early mammaliaforms (e.g., *Morganucodon*, *Megazostrodon*, *Haldanodon*; [32, 36]), and multituberculates [93, 100]. In the spalacotheriids, the ulnar condyle is bulbous anteriorly but flattened posteriorly, to form an incipient trochlea [34, 39]. In *Gobiconodon*, *Vincelestes* and therians, the trochlea is grooved instead of bulbous [33]. The capitulum, or radial condyle, is located laterally to the ulnar condyle. The capitulum is hemispherical and bigger than the ulnar condyle in anterior view. The capitulum is confined to the anterior and ventral aspect of the distal end, as seen in other non-mammaliaform cynodonts. The condyles are close and separated from each other only by a narrow intercondylar groove. Posteriorly, the olecranon fossa is very shallow as in non-mammaliaform cynodonts and early mammaliaforms [16, 19, 36], contrasting with the deep olecranon fossa seen in multituberculates, *Vincelestes* and therians [100].

**Fig 4:**
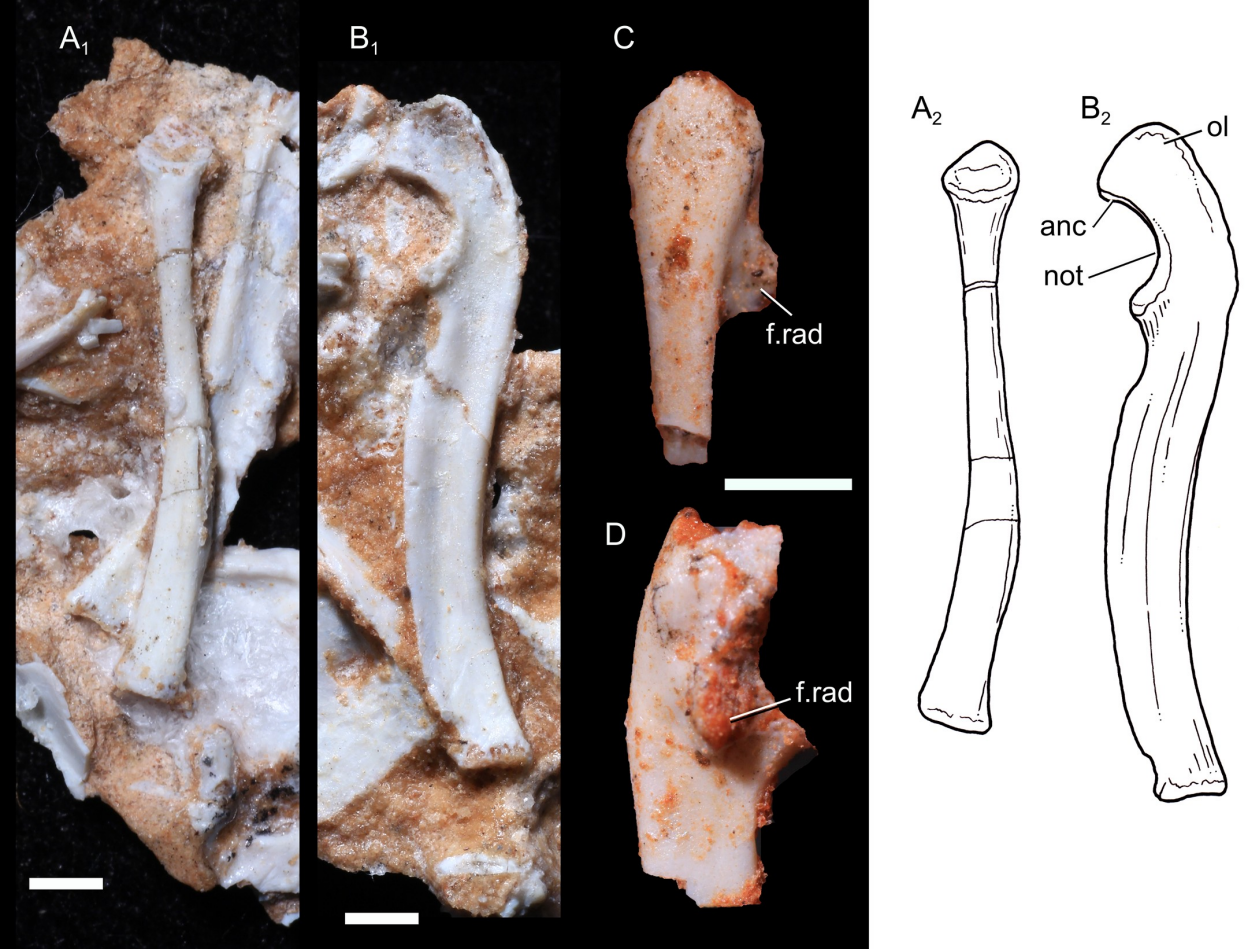
Forearm of *Brasilodon quadrangularis*. **A**, photography (1) and drawing (2) of the left radius (UFRGS-PV-1043-T), in medial view; **B**, photography (1) and drawing (2) of the right ulna (UFRGS-PV-1043-T), in medial view; **C**, photography of the right ulna (UFRGS-PV-0765-T), in posterior view; **D**, same material, in lateral view. **Abbreviations: anc**, anconeal process; **f.rad**, facet for the radial head; **not**, notch for the ulnar condyle; **ol**, olecranon. Scale bar = 2 mm.

**Radius:** The left radius UFRGS-PV-1043-T is complete and well-preserved, but it is embedded in the rock matrix, leaving the lateral surface unavailable (Fig 4A). The radius is a slender and elongate bone, with a length of 14.0 mm. Its morphology is similar to that of didelphid marsupials, although the prominent bicipital tuberosity in therians is not observed in *B*. *quadrangularis*. As in tritylodontids, *Morganucodon*, and didelphids, the radius of *B*. *quadrangularis* has a sigmoidal shape with the distal half slightly curved posteromedially to facilitate its crossing over the anterior aspect of the ulna. Proximally, the shaft is circular in cross-section and is nearly rectangular in the distal end, flattened and transversally widened. Distally, the medial edge of the shaft becomes prominent. The head of the radius is nearly circular, and consists of a circular and concave capitular facet, rimmed by a bulbous lip. The posterolateral rim extends more proximally than the anteromedial one, resulting in an articular facet inclined slightly anteromedially. A small articular facet for the radial notch of the ulna is present posteromedially to the proximal end. There is no distinctive radial tuberosity for the insertion of the *M*. *biceps brachii* on the radius. The distal articular surface is damaged but shows a concave and more rectangular morphology.

**Ulna:** The right ulna of UFRGS-PV-1043-T is well-preserved but is embedded in the rock matrix with only medial surface visible (Fig 4B). In other prozostrodontians, the medial and lateral surfaces of the bone are almost similar and the only dissymmetry is the facet for the contact with the capitulum, located on the lateral side [16, 19]. As this facet is not observed, the ulna is considered to be the right one. The shaft of the left ulna is also preserved and embedded in the sediment, but the articular facets are lost and only the lateral surface is visible. The right ulna of the specimen UFRGS-PV-0765-T lacks the distal midshaft and the olecranon, but most part of the proximal articular facet is available (Fig 4C and 4D). The right ulna of the UFRGS-PV-1043-T is 16.5 mm long. It is a slender bone with a sigmoidal shape in medial view. In anterior view, the shaft is narrow. The sigmoidal shape and the mediolateral narrowness of the shaft are common features in most mammals, but are also in most non-mammaliaform cynodonts, as *Galesaurus*, *Thrinaxodon*, *Cynognathus* [13], *Massetognathus* [12], *Andescynodon* [38], *Pascualgnathus*, *Trucidocynodon*, tritylodontids, and the mammaliaform *Morganucodon* [32]. The anterior and posterior margins are thickened, enclosing one longitudinal groove along the medial surface of the ulna. This groove extends from the proximal extremity to the distal one and represents the area of attachment for extensor muscles. A similar longitudinal groove is present along the lateral surface and is interpreted as for the origin of flexor muscles.

The olecranon is well-developed and projected anteriorly. Among non-mammaliaform cynodonts, only *Trucidocynodon*, *B*. *quadrangularis*, and tritylodontids have ossified olecranon processes [19, 98–99], and most basal cynodonts had probably cartilaginous processes, as this structure is not preserved in none of early taxa [12–13, 40, 81, 85, 87, 95]. Measured from the center of the semilunar notch, the length of the olecranon represents slightly less than 20% of the total ulnar length. Similar proportions are found in the basal probainognathian *Trucidocynodon* and the early mammaliaform *Megazostrodon*, whereas in the tritylodontid *Kayentatherium* and *Haldanodon*, the olecranon represents respectively more than 30% and 47% of the ulnar length [16, 36]. The olecranon process is wider transversally than the rest of the shaft. The notch for the ulnar condyle of the humerus is large and remarkably semicircular. The proximal end of this notch is situated more laterally than the more distal region. The anconeal process (olecranon beak) is reduced. This condition is seen in other non-mammaliaform cynodonts and *Haldanodon* [101], whereas a more prominent anconeal process is present as a crest-like structure in *Morganucodon*, multituberculates, spalacotheriids, *Vincelestes* and therians, demarcating the olecranon from the semilunar notch [39, 100]. The distal portion of this notch is anteriorly prominent relative to the ulnar shaft. A distinct facet for the radial head is developed distal to the facet for the ulnar condyle. The distal articular facet is not preserved.

#### Pelvic girdle and hindlimb

**Fig 5:**
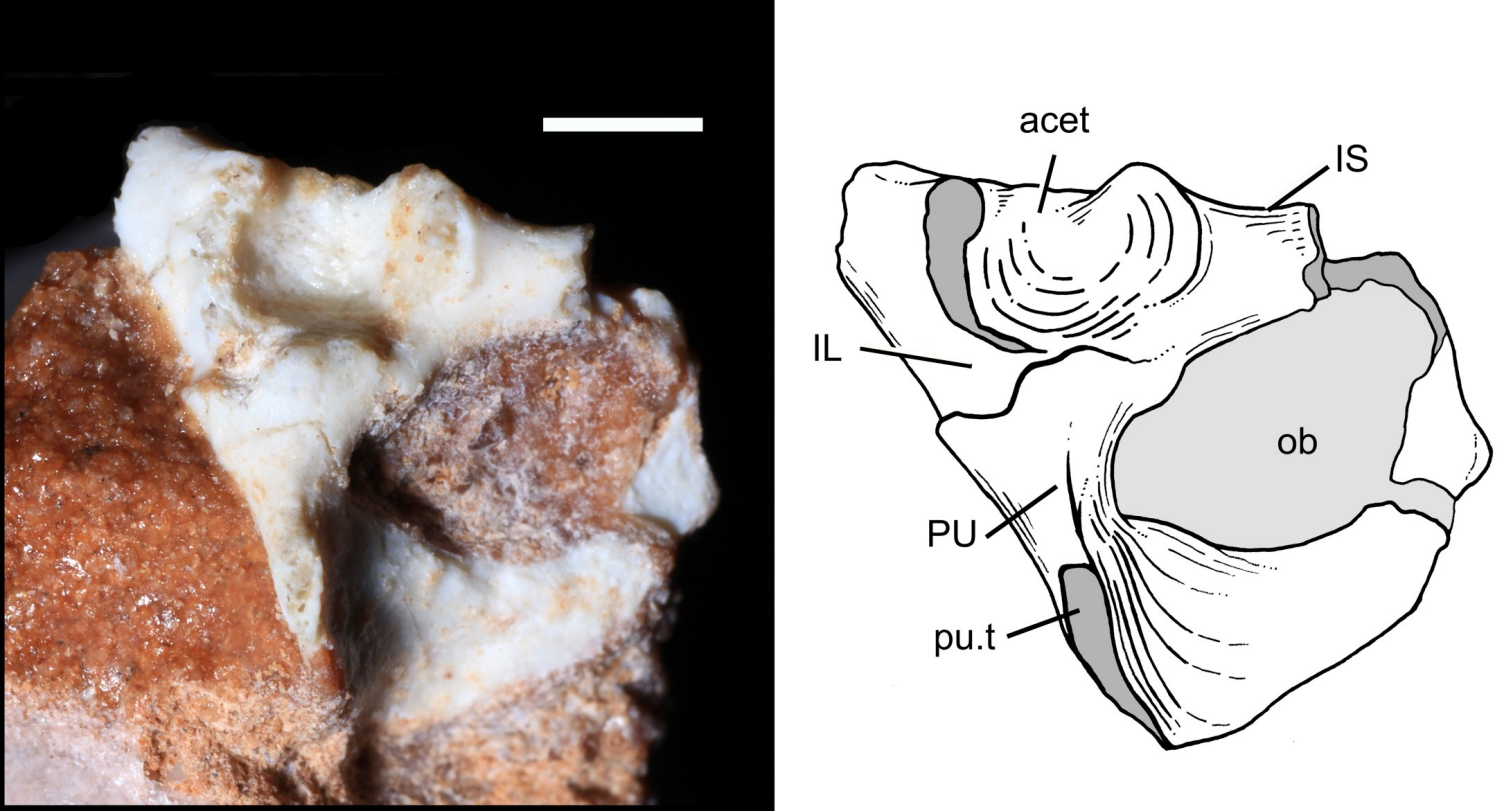
Photography and drawing of the left acetabulum of *Brasilodon quadrangularis* (UFRGS-PV-1043-T), in lateral view. **Abbreviations: acet**, acetabulum; **IL**, ilium; **IS**, ischium; **ob**, obturator foramen; **PU**, pubis; **pu.t**, pubic tuberosity. Scale bar = 2 mm.

The pelvic girdle is known from the complete left acetabulum, the complete left pubis and incomplete left ilium and ischium of the specimen UFRGS-PV-1043-T (Fig 5). The pelvis of *B*. *quadrangularis* differs considerably from those of non-mammaliaform cynodonts but resembles those of extant therians. The acetabulum is a deeply concave and spherical socket, facing laterally. As in most mammaliaforms, all three portions are fused together, without any sutures. By contrasts, in non-mammaliaform cynodonts with completely known acetabulum (e.g., *Pascualgnathus*, *Luangwa*, *Scalenodon*), the articulating faces of the three bones are more distinctly separated from one another by changes in angles, and the acetabulum is ovoidal and elongated along the anteroposterior axis. All of the three component bones contribute to moderately developed buttresses, extending laterally to the main plane of the pelvis. The supraacetabular iliac, ischial, and pubic buttresses are highly distinct, spaced 2–3 mm apart. The gap between the buttresses was presumably filled by fibrocartilage in life. This condition is also observed in tritylodontids, multituberculates [100, 102–104] and *Vincelestes*, but differs from the acetabulum of extant therians, which have a continuous bony rim (excepting the acetabular notch), without any dorsal emargination.

**Ilium:** Although the blade is not completely preserved in the specimen UFRGS-PV-1043-T, the iliac blade of *B*. *quadrangularis* appears to project anterodorsally from the acetabular region in life rather than anteriorly. A similar orientation is observed in *Galesaurus*, *Menadon* [82], *Pascualgnathus*, *Chiniquodon* [87], *Prozostrodon*, *Therioherpeton*, tritylodontids [16, 99, 104], early mammaliaforms and mammals [32]. Considering the morphology of the iliac neck, the postacetabular portion of the blade was reduced in *B*. *quadrangularis*, corresponding to a small prominence, as in *Prozostrodon* [43] and tritylodontids [16, 104], or completely absent as in *Therioherpeton* and the early mammaliaforms (e.g., *Eozostrodon*, *Megazostrodon*, *Erythrotherium*; [32]). In other non-mammaliaform cynodonts, the postacetabular region of the blade is well-developed [12–13, 38, 80, 82, 86–87, 95, 105].

The supracetabular buttress of the ilium is projected laterally to the main plane of the pelvis. It lies anteriorly rather than dorsally to the acetabulum, leaving the dorsal region of the acetabulum open. This condition is present in *Aleodon* [80], traversodontids [12, 82, 86], *Prozostrodon* [43], and tritylodontids [104]. By contrast, in the basal cynodonts *Procynosuchus* [14], *Galesaurus*, and *Cynognathus*, the iliac buttress has a dorsal position. As in therians, the iliac facet of the acetabulum is semi-circular and facing laterally and posteroventrally rather than ventrally. The iliac portion of the acetabulum is facing mainly ventrally in basal cynodonts [13–14, 84], *Pascualgnathus* and *Trucidocynodon* [18].

**Ischium:** Only the anteromedial margin of the ischial blade of UFRGS-PV-1043-T is preserved. This margin is semi-circular and forms an extended and oval obturator foramen with the pubis. In *Luangwa* [42], *Andescynodon* [38], *Menadon* [82], *Exaeretodon* [86], *Chiniquodon* [87, 106], *Therioherpeton*, *Trucidocynodon*, tritylodontids [98, 104] and *Morganucodon* [32], the obturator foramen is large, as seen in mammals. The basal cynognathians (e.g., *Cynognathus* and *Diademodon*) and *Pascualgnathus* have a smaller foramen with the anteromedial margin of the ischium projected more medially, as in basal therapsids [13]. Posteriorly to the obturator foramen, the ischial plate becomes narrow and meets the posterior edge of the pubic plate, anteromedially to the obturator foramen. The buttress is well-marked and located posteriorly to the acetabulum. The acetabular facet of the ischium is concave and facing anteriorly and laterally.

**Pubis:** The pubis of UFRGS-PV-1043-T is almost complete and well-preserved (Fig 5), other than the head of the pubis is slightly damaged. Below the head, the pubis constricts to form a short and twisted neck ending with a pubic tuberosity, located ventral to the acetabulum. Distally, the neck is everted and projected medially and posteriorly. The pubic tuberosity is projected ventrally to the acetabulum and no part of the bone is projected anteriorly to the acetabulum. In *Luangwa*, *Pascualgnathus*, other non-mammaliaform probainognathians, living therians and all other Mesozoic mammal lineages with known pelves (including *Zhangheotherium*, *Maotherium*, *Vincelestes*; [43, 107–109]), the pubic tuberosity is located ventrally to the acetabulum. The pubic tuberosity is located anteriorly to the acetabulum rather than ventrally in basal epicynodonts, living monotremes and *Akidolestes* [39]. The pubic blade of *B*. *quadrangularis* is flat and relatively expanded transversally. The medioventral margin of the blade is straight and represents certainly the symphysis with the opposite pubis. The dorsal margin is concave, forming the obturator foramen with the ischium. The acetabular facet is facing dorsally.

**Fig 6:**
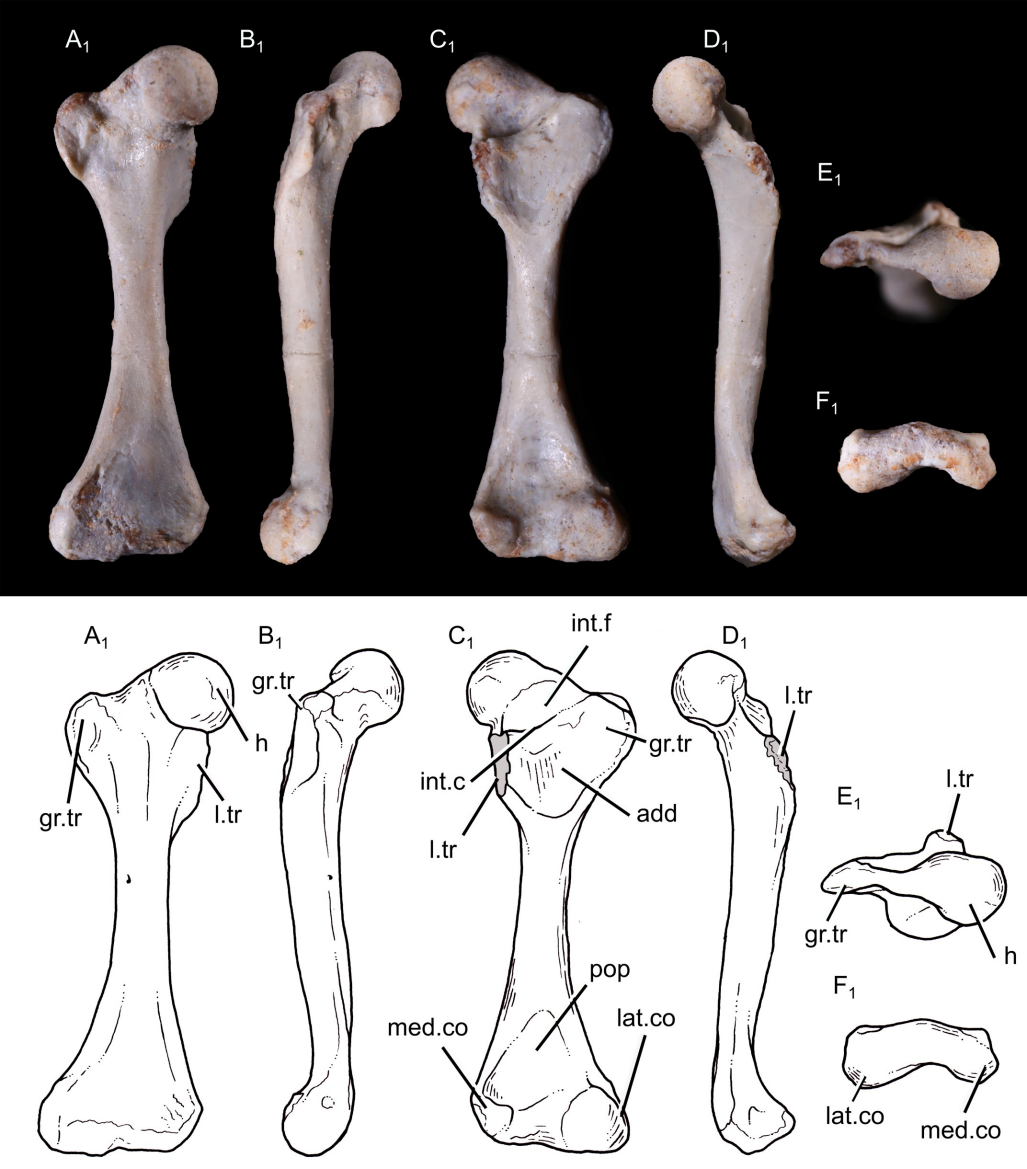
Photography (1) and drawing (2) of the right femur of *Brasilodon quadrangularis* (UFRGS-PV-1043-T). **A**, right femur in anterior view; **B**, right femur in lateral view; **C**, right femur in posterior view; **D**, right femur in medial view; **E**, right femur in proximal view; **F**, right femur in distal view. **Abbreviations: add**, fossa for hip adductor muscles; **gr.tr**, greater trochanter; **h**, femoral head; **int.c**, intertrochanteric crest; **int.f**, intertrochanteric fossa; **lat.co**, lateral condyle; **l.tr**, lesser trochanter; **med.co**, surface for the medial condyle; **pop**, popliteal fossa. Scale bar = 3 mm.

**Femur:** The right femur of UFRGS-PV-1043-T is completely preserved (Fig 6) and the left one is represented only by the proximal end, embedded in the matrix. The bone is described with the shaft held vertically, with a mammalian orientation. The femur is 15.9 mm long. The shaft is straight except the most proximal part which is projected anteriorly. It is a condition similar to that of other non-mammaliaform cynodonts and early mammaliaforms (e.g., [13, 32, 36]). The proximal portion of the shaft is only slightly dorsally projected in living mammals [13].

*Brasilodon quadrangularis* has a hemispherical femoral head [27] with a morphology similar to *Tritylodon* [19], early mammaliaforms [32, 36] and most mammals (e.g., [39, 100]). Most tritylodontids have a hemi-ovoid head, elongated along the posterolateral-anteromedial axis [16, 98, 104] and more basal non-mammaliaform cynodonts have a flattened and bulbous femoral head, with less extensive dorsoventral curvature. The femoral head of *B*. *quadrangularis* is located medially on the proximal end and projects somewhat anteriorly by virtue of the anterior bowing of the proximal part of the shaft. The femoral head of *B*. *quadrangularis* is set off from the shaft by a constriction although its lateral margin is not clearly distinct from the rest of the proximal end. A short femoral neck is also observed in tritylodontids [19, 89, 98] and mammaliaforms but is absent in other non-mammaliaform cynodonts. The head projects strongly medially with an angle of about 60° relative to the longitudinal axis of the femur. The medial projection seen in *B*. *quadrangularis* contrasts with the femoral head of ‘pelycosaurs’, located on line with the axis of the shaft [13, 42]. Among other non-mammaliaform prozostrodontians, this angle is variable but never exceeds 45°. The strong medial projection of the femoral head as *B*. *quadrangularis* is also observed in multituberculates [37, 93, 100] and the spalacotheriids [108], although the neck is shorter in *B*. *quadrangularis* than in these mammals. The fovea capitis femoris seen in morganucodontids, multituberculates and extant mammals [35, 100] is absent in *B*. *quadrangularis*, as in all other non-mammaliaform cynodonts.

The well-developed greater trochanter is a distinct tuberosity projected laterally and connected to the femoral head by a narrow ridge. By contrast, the femoral head is confluent with the greater trochanter in basal epicynodonts [84, 110], traversodontids [12, 38, 86, 91], *Trucidocynodon*, *Chiniquodon* [87, 106], *Prozostrodon*, *Therioherpeton*, and *Irajatherium*. In tritylodontids and early mammaliaforms, the greater trochanter is separated from the head of the femur by a slight notch, as in mammals [16, 97– 98, 104]. The apex of the greater trochanter reaches the midline level of the femoral head. This condition differs from multituberculates and *Zhangheotherium*, which have a greater trochanter reaching well above the level of the femoral head, but also from most basal eucynodonts (e.g., *Cynognathus*, *Diademodon*, *Cricodon*, *Andescynodon*) which have a less prominent greater trochanter, not reaching the level of the femoral head [38, 40, 100, 110–111].

The lesser trochanter is slightly eroded. It is a short crest, separated from the head and located close to the intertrochanteric fossa. The lesser trochanter is projected medially and remains visible in anterior view. This is a derived condition found in several non-mammaliaform cynodonts (e.g., *Prozostrodon*, *Therioherpeton*, *Trucidocynodon*, *Irajatherium*, *Andescynodon*, tritylodontids; [16, 38, 43, 98, 104, 112] and in early mammaliaforms (*Morganucodon*, *Megazostrodon*, *Haldanodon*), but the later differs from *B*. *quadrangularis* having a trochanter located proximally, reaching the level of the femoral head [32, 36]. In basal cynodonts, as *Procynosuchus* and *Cricodon*, the lesser trochanter is projected posteriorly [110, 14] and in *Thrinaxodon*, *Diademodon*, *Chiniquodon* [106], *Boreogomphodon* [85, 91], *Menadon* [82], *Massetognathus* [12], *Exaeretodon* [86], *Pascualgnathus*, and *Santacruzodon* [113] the lesser trochanter has a posteromedial position but is not projected as medially as in *B*. *quadrangularis* and is not visible in anterior view. As in other non-mammaliaform cynodonts, a third trochanter is absent.

The intertrochanteric fossa is narrow and deep instead of being circular as in other non-mammaliaform cynodonts. It is located on the posterior surface of the shaft, immediately distally to the femoral head. The most characteristic feature is the prominent crest, forming the distal margin of the intertrochanteric fossa and connecting the two trochanters. An intertrochanteric crest is observed in most living therians but is absent in other non-mammaliaform cynodonts, early mammaliaforms and most Mezosoic mammal lineages (e.g., *Akidolestes*, *Vincelestes*). Distally to the crest, a triangular fossa extends until the distal extremity of the lesser trochanter and represents the area of insertion for hip adductor muscles. This fossa is shallower and wider than the trocantheric fossa. It is observed in most non-mammaliaform cynodonts, *Vincelestes* and living therians as *Didelphis*, but is lacking in *Prozostrodon*, *Tritylodon* [19] and *Oligokyphus*.

The shaft of the femur is straight. It is approximately square in cross-section but becomes more rectangular distally, being compressed anteroposteriorly. The distal extremity of the femur gradually expands transversally. This extremity reaches the same width as the proximal one. The proximal and distal ends are also broader than the shaft in other non-mammaliaform cynodonts, *Haldanodon*, *Gobiconodon*, and *Akidolestes* [33, 36, 39]. The proximal and distal ends are not such expanded transversally in *Morganucodon* [32] and are only slightly broader than the shaft in multituberculates, *Zhangheotherium*, *Vincelestes* and extant therians [34, 37, 93, 100]. Distally, a well-defined and triangular popliteal fossa is present on the posterior surface, near the medial and lateral condyles. Both condyles are facing mainly posteriorly. The medial condyle is narrower than the lateral one, being transversally compressed, as seen in most non-mammaliaform cynodonts (e.g., [12–13, 15–16, 104]), early mammaliaforms [35–36], Mesozoic mammal lineages (e.g., [34, 108]) and didelphids.

**Fig 7:**
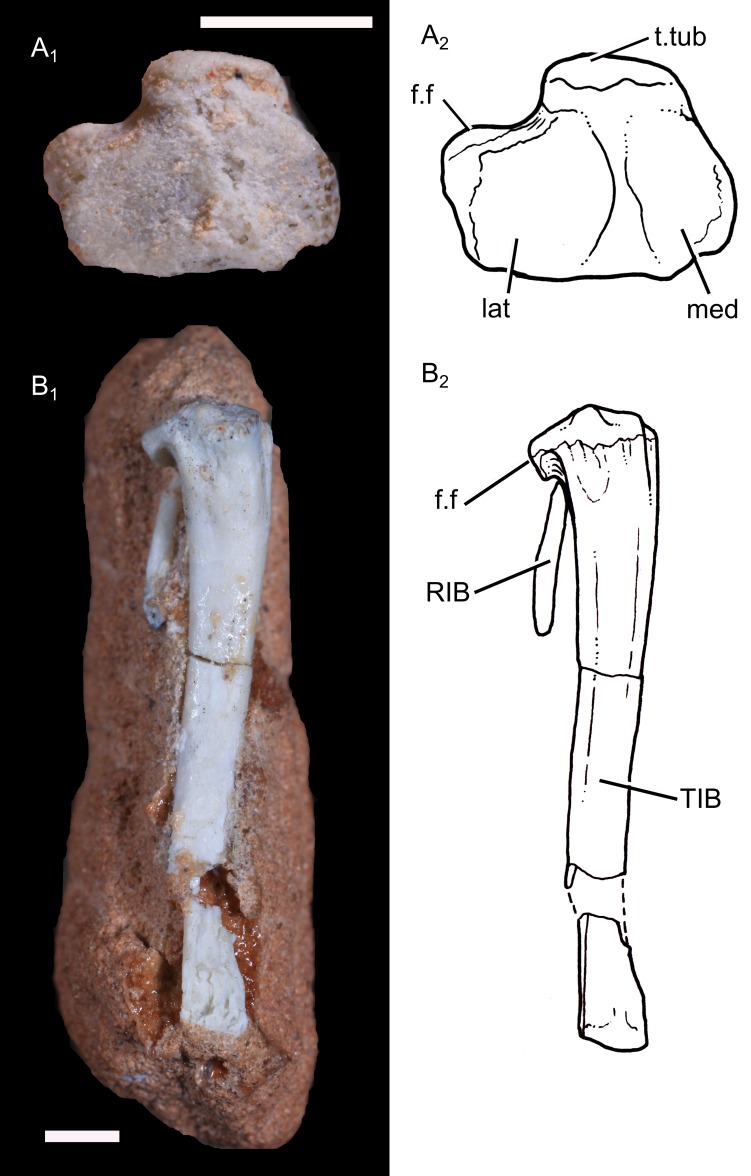
Photography (1) and drawing (2) of the left tibia of *Brasilodon quadrangularis* (UFRGS-PV-1043-T). **A**, in proximal view; **B**, in posteromedial view. **Abbreviations: f.f**, facet for the fibula; **lat**, facet for the lateral femoral condyle; **med**, facet for the medial femoral condyle; **RIB**, fragment of rib; **TIB**, tibia; **t.tub**, tibial tuberosity. Scale bar = 2 mm.

**Tibia:** The left tibia of UFRGS-PV-1043-T is well-preserved and almost complete, except the damaged shaft and the distal epiphysis (Fig 7). The bone has a preserved length of 16.1 mm. Its morphology, including the proximal articular surface, is similar to that of living therians. The proximal end is compound of two oval-shaped and slightly concave facets for the femoral condyles. They are similar in anteroposterior length, but the medial facet is narrower transversally than the lateral one. The two facets are separated by a low ridge. A projection located on the anterior margin of the articular surface represents the tibial tuberosity for the insertion of patellar ligament. The cnemial crest extends distally from the tibial tuberosity and runs along the anterolateral margin of the shaft. As this surface of the tibia is embedded in the matrix, the length of the crest is uncertain. A strong process is located on the lateral margin for the contact with the fibula.

The shaft is slender. The diameter of the shaft decreases gradually distally and becomes more flattened. Although the proximal shaft is very slightly curved medially, the tibia of *B*. *quadrangularis* has a straight shaft, contrasting with most non-mammaliaform cynodonts which have a medially bowed tibia [12, 14, 84–86]. The shaft is straight in the tritylodontid *Beinotheroides* [99] and *Prozostrodon*.

**Fig 8:**
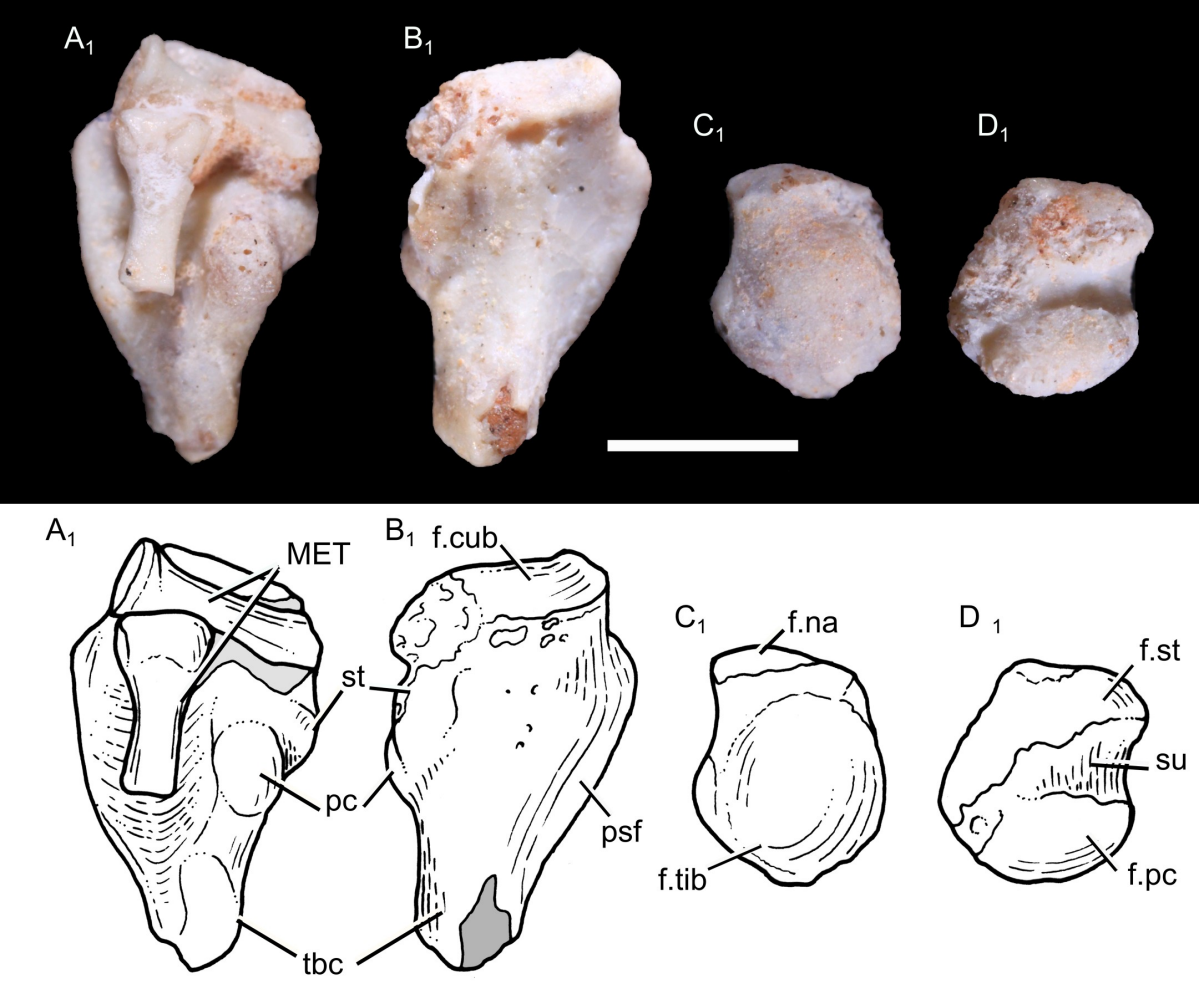
Photography (1) and drawing (2) of the calcaneum and astragalus of *Brasilodon quadrangularis* (UFRGS-PV-1043-T). **A**, left calcaneum, in dorsal view; **B**, left calcaneum, in plantar view; **C**, left astragalus, in dorsomedial view; **D**, left astragalus, in lateroplantar view. **Abbreviations: f.cub**, facet for the cuboid; **f.na**, facet for the navicular; **f.pc**, facet for the proximal facet of the calcaneum; **f.st**, facet for the sustentaculum tali; **f.tib**, facet for the tibia; **MET**, metatarsus; **pc**, proximal facet of the calcaneum for the astragal; **psf**, peroneal shelf; **st**, sustentaculum tali; **su**, sulcus of the astragalus (tarsal sinus); **tbc**, tuber calcis. Scale bar = 2 mm.

**Tarsus:** The left calcaneum and astragalus of UFRGS-PV-1043-T were found together but not totally articulated. The calcaneum is dorsoplantarly compressed and transversely broad (Fig 8A and 8B), with a peroneal shelf projected laterally to the calcaneal body, as in tritylodontids, *Morganucodon* and *Fruitafossor* [32]. By contrast, *Zhangheotherium*, multituberculates and therians have a transversely narrow calcaneum [111, 114]. In lateral view, it is slightly arched, being concave plantarly and convex dorsally. The tuber calcis is prominent and projected posteroplantarly, as in other eucynodonts [13, 18, 85] whereas the calcaneum of *Galesaurus* and *Thrinaxodon* are oval, with no ossified or developed tuber calcis [13, 84]. Dorsally, the lateral and medial margins of the calcaneum are inflected and enclose a wide fossa, for the passage of tendons of digit extensor muscles. On the medial side of the calcaneum is a stout and bulbous process, lying approximately on the transverse midline of the bone. It is directed dorsomedially and represents the posterior (proximal) facet for the contact with the lateral surface of the astragalus. Anteroplantar to the proximal process is another distinct process, the sustentaculum tali, projected medially. The sustentaculum tali articulated with the plantar surface of the astragalus. It represents an incipient morphological reorientation of the two proximal tarsi, toward the mammalian condition, in which the astragalus overlaps the calcaneum. This condition is observed in some eucynodonts [13, 18, 85], but no distinct sustentaculum tali can be observed in basal epicynodonts (e.g., *Thrinaxodon*, *Galesaurus*), suggesting it was cartilaginous or not developed yet [13]. The sustentaculum tali and the posterior process are separated by a calcaneal sulcus. A groove is located plantar to the processes, oriented anteroposteriorly, probably for the passage of a long flexor tendon. The rectangular, nearly flat cuboid facet faces anteriorly.

The astragalus is a hemispherical bone (Fig 8C and 8D), with a bulbous dorsomedial surface and a flat lateroplantar surface, as seen in other non-mammaliaform cynodonts. It is notably smaller than the calcaneum. A large, oval facet lies on the posterior half of the lateroplantar surface for the proximal process of the calcaneum. On the anterior half is another facet for the sustentaculum tali. The two facets have approximately the same size and are separated by a deep, concave sulcus, which represent the astragalar half of the tarsal sinus. The dorsomedial surface represents the articulation with the tibia. Anteriorly, the astragalus is constricted to form a very short but distinct astragalar head, bearing a slightly convex facet for the articulation with the navicular. In therians, the neck separating the astragalar head from the body is longer [115–118].

**Fig 9:**
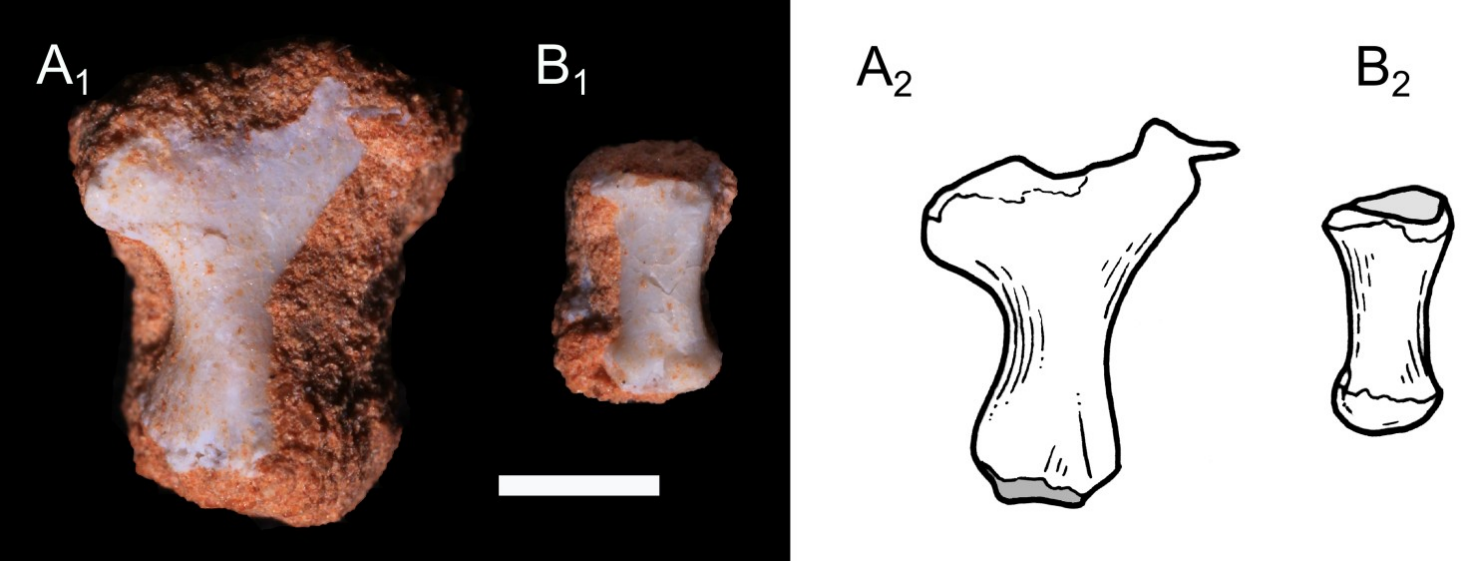
Photography (1) and drawing (2) of the metapod and phalange of *Brasilodon quadrangularis* (UFRGS-PV-0765-T). **A**, fragment of a metapod; **B**, intermediate phalange, in dorsal view. Scale bar = 2 mm.

**Metapods and phalanges:** Two partial metatarsals are preserved with the calcaneum of UFRGS-PV-1043-T (Fig 8A). The first one, representing a proximal end, is ovoid and bears a concave and circular fossa. The second one represents a distal end and bears two distinct and symmetric condyles. Another fragment of a possible metapod is also preserved in UFRGS-PV-0765-T (Fig 9A). The proximal portion is broadly expanded transversally and the shaft is narrow. Moreover, a few partial phalanges are preserved in UFRGS-PV-1043-T and UFRGS-PV-0765-T. One phalange is almost complete (Fig 9B). It is short and represents probably the middle element of the digit. Its proximal surface is concave and its distal end is marked by two small and distinct condyles.

## Discussion

**Fig 10:**
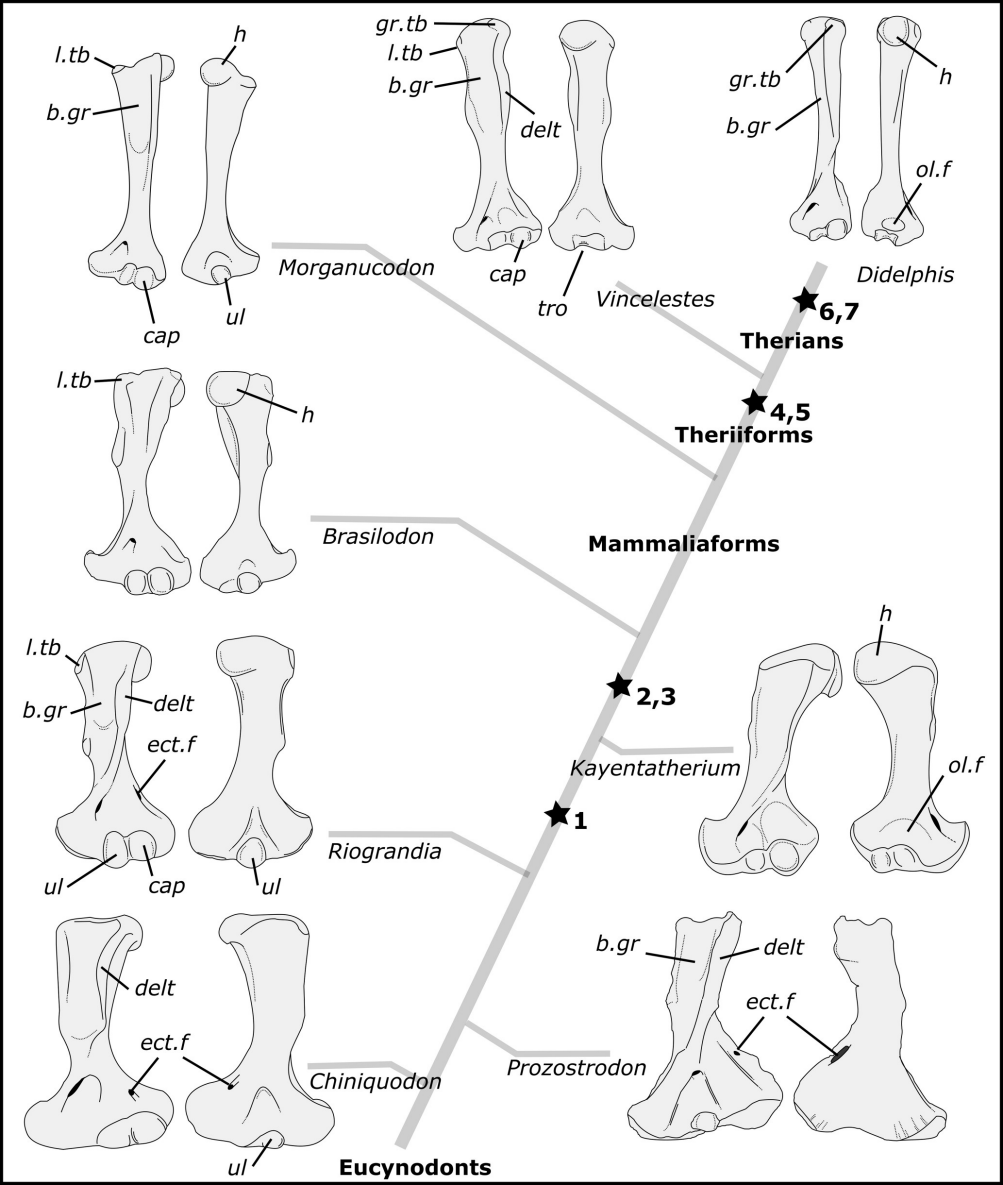
Main evolutionary patterns of the humeral structures through the probainognathian evolution. The left humeri are represented in anterior and posterior view. **1**, loss of ectepicondylar foramen; **2**, hemispherical humeral head; **3**, distinct and bony greater tubercle; **4**, posteriorly flat trochlea (instead of bulbous ulnar condyle) with deep olecranon fossa; **5**, less anteriorly expanded deltopectoral crest with narrow bicipital groove; **6**, lesser tubercle smaller than the greater one; **7**, posteriorly facing humeral head.. The ectepicondylar foramen is also absent in *Boreogomphodon*, *Trucidocynodon* and *Probainognathus*, but it is not represented in this phylogeny. The ulnar condyle is bulbous posteriorly in multituberculates, but it is not represented in this phylogeny. **Abbreviations: b.gr**, bicipital groove; **cap**, capitulum; **delt**, deltopectoral crest; **ect.f**, ectepicondylar foramen; **gr.tb**, greater tubercle; **h**, humeral head; **l.tub**, lesser tubercle; **ol.f**, olecranon fossa; **tro**, trochlea; **ul**, ulnar condyle.

**Fig 11:**
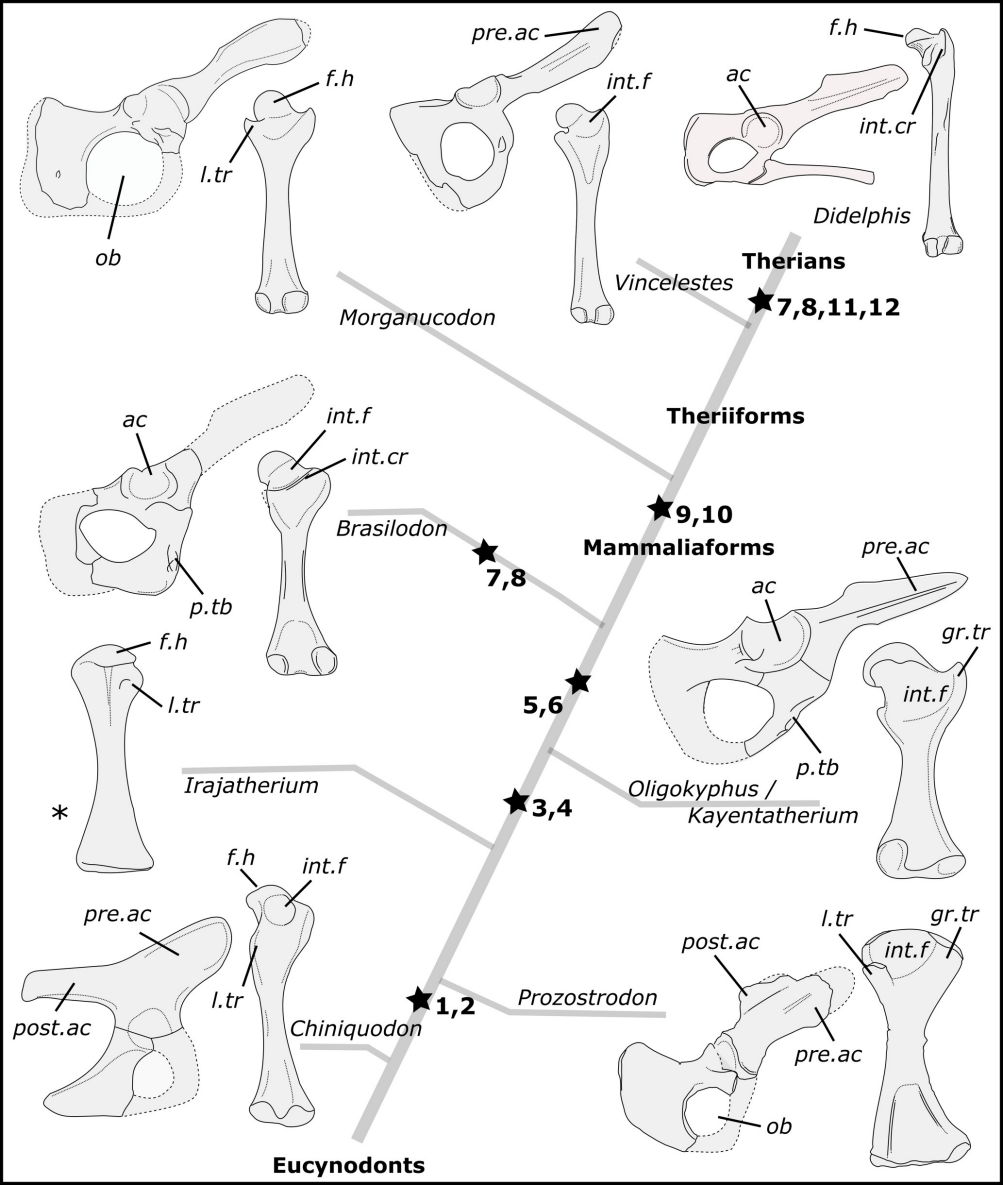
Main evolutionary patterns of the pelvic and femoral structures through the probainognathian evolution. The right pelvis and femora are respectively represented in lateral and posterior view (with exception of the femur of *Irajatherium*, which is represented in anterior view*). **1**, reduced postacetabular region of the iliac blade; **2**, medial projection of the lesser trochanter; **3**, distinct greater trochanter; **4**, head set-off from the shaft by a short femoral neck; **5**, confluent acetabular facets of the three bones composing the acetabulum; **6**, hemispherical femoral head; **7**, narrow intertrochanteric fossa instead of wide and circular; **8**, well-marked intertrochanteric crest; **9**, presence of the fovea capitis; **10**, narrow distal end of the femur; **11**, rim of the acetabulum without dorsal emargination; **12**, femoral head strongly projected medially and long femoral neck. The hemispherical femoral head is only present in *Tritylodon* among Tritylodontidae, but it is not represented in this phylogeny. **Abbreviations: ac**, acetabulum; **f.h**, femoral head; **gr.tr**, greater trochanter; **int.cr**, intertrochanteric crest; **int.f**, intertrochanteric fossa; **l.tr**, lesser trochanter; **ob**, obturator foramen; **post.ac**, postacetabular region of the iliac blade; **pre.ac**, preacetabular region of the iliac blade; **p.tb**, pubic tuberosity.

The humerus of *B*. *quadrangularis* bears a combination of plesiomorphic and derived features. For example, posterolaterally facing humeral head, lesser tubercle larger than the greater one, long and anteriorly expanded deltopectoral crest, wide bicipital groove, teres major tuberosity, wide distal humeral extremity, shallow olecranon fossa and bulbous ulnar condyle are features seen in other non-mammaliaform cynodonts. On the other hand, ventrally oriented scapular glenoid facet, lack of ectepicondylar foramen and osseous olecranon process are similar to the condition only present in more advanced prozostrodontians (e.g., tritylodontids), and hemispherical humeral head and distinct and ossified greater tubercle are present in mammaliaforms (Fig 10). The pelvic girdle and the femur of *B*. *quadrangularis* reveal derived feature as reduced postacetabular region of the iliac blade, large obturator foramen, no part of the pubis projecting anteriorly to the acetabulum, confluent parts of the acetabulum, hemispherical and medially projected femoral head, head set-off the femoral shaft, distinct greater trochanter, medially projected lesser trochanter, and prominent intertrochanteric crest, similar to early mammaliaforms (e.g., *Morganucodon*) or therians (Fig 11).

### Limb posture of *Brasilodon quadrangularis*

#### Orientation of the glenoid

The scapular portion of the glenoid of this taxon faces ventrally but the coracoid and the procoracoid of *B*. *quadrangularis* remain unknown, leaving the complete orientation of the glenoid ambiguous. Moreover, if a laterally facing glenoid can prevent an adducted position of the humerus, a ventrally facing glenoid does not necessarily imply an erect posture [119]. Living monotremes have a sprawling posture, but a major part of their glenoid fossa (formed by the scapula) faces ventrally, with only a small coracoid portion facing laterally.

### Torsion of the humeral shaft

The torsion (or twisting) of the humerus is regarded as a very important character to determinate sprawling or erect posture in fossil tetrapods [120–121]. Humeral torsion is indicative of sprawling posture and occurs in terrestrial tetrapods which have an abducted humerus (e.g., monotremes, lacertilians) whereas it is absent in therians which have an erect posture [90]. The torsion measured on the humerus of *B*. *quadrangularis* is weaker than in monotremes, most non-mammaliaform cynodonts and Mezosoic mammal lineages, but remains stronger than in therians, indicating at least a semi-sprawling posture of the forelimb.

### Importance of the adductor musculature

A long deltopectoral crest can increase the efficiency of the *M*. *pectoralis*. In basal tetrapods and reptiles, the *M*. *pectoralis* is mainly an adductor of the forelimb and plays a substantial role in maintenance of limb posture, elevating the body from the ground in animals which have an abducted humerus [13, 51, 122]. By contrast, the adductor function is limited to *M*. *pectoralis superficialis* in generalized therians, and *M*. *pectoralis profundus* is a powerful retractor, with a reduced role in posture [64, 66, 123]. The length of the crest and its great anterior projection seen in *B*. *quadrangularis* and all non-mammaliaform cynodonts suggest that the adductor function of the *M*. *pectoralis* had an important role in posture and the humerus was held in a sprawling or semi-sprawling position.

The bicipital groove of the humerus is well-marked and deep proximally in *B*. *quadrangularis*. It is broad, providing a wide area for the *M*. *coracobrachialis*. This muscle could assist the *M*. *pectoralis* to adduct the humerus, and sustain the body. In contrast, the bicipital groove is narrow in therians with parasagittal posture, as only the tendon of the *M*. *biceps brachii caput longum* passes along the groove [90].

In *B*. *quadrangularis*, the lesser tubercle is bigger than the greater tubercle and protrudes strongly medially, increasing the lever arm of *M*. *subscapularis*. The larger transverse diameter of the lesser tubercle than of the greater tubercle reflects the importance of this humeral adductor muscle. This is typical for animals with abducted forelimbs, such as lacertilians, monotremes and some fossorial mammals with sprawling (talpids) or semi-sprawling (chrysochlorids) posture [90]. In therians with parasagittal limb, the lesser tubercle is smaller and narrower than the greater tubercle.

### Ectepicondylar foramen

The ectepicondylar foramen present in early synapsids and reptiles is for the passage of radial nerve [124]. The absence of this foramen in *B*. *quadrangularis* and mammaliaforms indicates presumably a change in position of origins of extensor muscles of the hand, which evolved a shift in the course of the radial nerve. According to Romer [125], this shift can be correlated with a position of the humeral distal end under the body. However, the absence of the ectepicondylar foramen in monotremes, which have a sprawling forelimb posture, argues against this hypothesis. This foramen has disappeared very early during the pre-mammalian evolution (within tritylodontids, *Brasilodon*, and early mammaliaforms), certainly before the adaptation to an erect posture.

### Ulnar condyle

All tetrapods with a primary sprawling posture have a condylar structure of the elbow joint. In lacertilians, the ulnar condyle is bulbous, to permit rotation of the forearm, which is necessary for sprawling locomotion [90, 126]. The ulnar condyle is confined only in anterior and distal view. This is consistent with the horizontal orientation of the humerus during the whole propulsive phase. As the elbow extension is relatively small [48], the condyles will be useless on the posterior part. In contrast, in therians, the ulnar condyle is cam-shaped, being flat anteriorly and overlapping the distal end until the posterior surface of the humerus [119, 127]. The intercondylar groove separating the ulnar and radial condyles is also enlarged. With the ulnar condyle, this groove forms the trochlea, which contains and embraces the motion of the elbow joint in a parasagittal plane [128–129].

The presence of a bulbous ulnar condyle (rather than a trochlea as in therians) suggests that the rotation of the forearm was still an important element of the locomotion of *B*. *quadrangularis*. The ulnar condyle of *B*. *quadrangularis* is compressed and extends until the posterior part of the humerus, indicating an incipient origin of the trochlear structure and wider flexion-extension capability of the elbow joint. It should be remembered, however, that such capability does not necessarily imply parasagittal locomotion and is observed in fossorial and semifossorial mammals, which secondarily acquired sprawling or semi-sprawling posture [94]. Moreover, in spite of the posterior extension of the ulnar condyle, the narrow intercondylar groove, and the reduced ulnar anconeal process of *B*. *quadrangularis* could not contain efficiently the motion in a parasagittal plane.

### Olecranon process

The olecranon process of the ulna is the area of insertion for the *M*. *triceps brachii* in all tetrapods (e.g., [64, 66]). This process is well-developed in *B*. *quadrangularis*, differing with most non-mammaliaform cynodonts, and indicating extension of the elbow was an important element of the locomotion of this cynodont. This condition differs from animals with sprawling locomotion [48, 126].

### Acetabulum and femoral head

The acetabulum and femoral morphologies of *B*. *quadrangularis* are very similar to those of living therians. The hemispherical femoral head permits a higher range of motion than the flattened head of ‘pelycosaurs’ synapsids or basal non-mammaliaform cynodonts, and could provide the same range of motion than early mammaliaforms and living therians [15, 27, 32]. The center of the head is not located in line with the axis of the shaft as in ‘pelycosaurs’, but is strongly projected medially, with an angle of 60° to the shaft, as in living therians. The position of the femoral head of *B*. *quadrangularis* indicates the shaft was held close to the parasagittal plane and a shift toward a more erect hindlimb posture. However, the short neck suggests a less erect posture than in living therians.

### Femoral condyles

Femoral condyles of *B*. *quadrangularis* are confined to the posterior aspects of the bone, and do not extend around the distal extremity. This feature can be found in mammals with either sprawling or parasagittal posture, but indicates a crouched posture, with a flexed lowerleg [100]. The asymmetrical knee joint in *B*. *quadrangularis*, with the medial condyle narrower than the smaller lateral one, can lead to several interpretations. First, it could suggest a sprawling hindlimb posture for this cynodont. The monotremes have an asymmetrical distal femur, and this configuration is necessary as their sprawling locomotion implies horizontal swinging of the femur, and thus rotation of the knee joint [108]. However, cineradiographic records of walking *Didelphis* and other non-cursorial mammals give some evidence of rotation at the knee during the propulsive movement [130]. The femur, oriented obliquely to the sagittal plane at the beginning of a step, abducts during the propulsive movement, whereas the lower leg, remains in a nearly sagittal plane. Such a change in relative orientation of the femur and tibia may be accommodated by a mechanism permitting rotation at the knee joint and thus an asymetric joint. Within didelphid marsupials, the more asymmetrical knee joint is associated with more habitually flexed knee (crouched position), for better climbing locomotor capability [131]. In contrast, more symmetric knee joint tends to be associated with the didelphid species with terrestrial habits [61].

### Hip musculature

The hip muscular organization changed considerably during the evolution of non-mammaliaform cynodonts (e.g., [43]). In ‘pelycosaur’ synapsids, the retraction was driven mainly by the *M*. *caudifemoralis*, which was assisted also by the posterior part of the *M*. *obturator externus* (homologue of the *M*. *pubo-ischio-femoralis externus* in non-mammalian tetrapods), as in early amniotes and reptiles [41, 132]. In ‘pelycosaurs’ and other basal synapsids, the *M*. *glutei* (homologue of the *M*. *iliofemoralis* in non-mammalian tetrapods) originated on the lateral surface of the posterior and middle parts of the iliac blade (Fig 8), as seen in living squamates and urodelans [41]. These areas were the most appropriate to the function of abduction and rotation of the femur about its long axis during sprawling locomotion [42]. In most non-mammaliaform cynodonts such as *Cynognathus* or *Chiniquodon*, the preacetabular portion of the iliac blade is extended anteriorly permitting the reorientation of the gluteal musculature, closer to mammals than to ‘pelycosaurs’. The posterior fibers of the *M*. *glutei* retained an abductor function but the most anterior fibers can pass dorsally and posteriorly to the center of rotation of the hip joint to insert on the greater trochanter, permitting the gluteus musculature to retract the hindlimb. The most anterior fibers could permit to increase the posterior extent to which the femur could retract compared to that of basal synapids [14]. Although incomplete, the ilium of *B*. *quadrangularis* was clearly more similar to derived non-mammaliaform cynodonts (e.g., *Prozostrodon* and tritylodontids) and therians than to the basal non-mammaliaform cynodont morphology, having a rod-like shaped iliac blade with a reduced postacetabular portion. The reduced postacetabular portion and the prominent and distinct greater trochanter observed in *B*. *quadrangularis* and tritylodontids suggest the mechanism of gluteus-driven retraction had replaced the reptilian *M*. *caudifemoralis* as the main retractor muscle of the leg.

In ‘pelycosaurs’, the *M*. *iliacus* (homologue of the *M*. *pubo-ischio-femoralis internus* in non-mammalian tetrapods) originated from the anterodorsal portion of the puboischial plate, as seen in basal amniotes and monotremes [41, 46, 56]. The *M*. *iliopsoas* could not insert on the internal trochanter taking account of the posterior position of this trochanter. The insertion of the *M*. *iliopsoas* (fusion of the *M*. *iliacus* and the *M*. *psoas major*) was more likely located on the anterior surface of the femur, as in basal amniotes [13, 133]. The anterior position of the pubic tuberosity and the posterior position of the lesser trochanter is observed in *Procynosuchus*, *Galesaurus*, *Diademodon* and *Cynognathus* and indicates that most basal cynodonts had similar muscular configuration to basal synapsids. In *B*. *quadrangularis* and other non-mammaliaform probainognathians, the *M*. *iliacus* originated from the ventral part of the lateral surface of the well-developed preacetabular portion of the iliac blade, as seen in *Didelphis* [61], as no part of the pubis is projected anteriorly to the acetabulum. The dorsal migration of the origin of the *M*. *iliacus* was accentuated by the projection of the iliac blade, which is more anterodorsal than anterior in prozostrodontians. In therians, the *M*. *iliopsoas* insert on the lesser trochanter [61, 65, 67] which protrudes medially. The medial projection of lesser trochanter seen in *B*. *quadrangularis* and most derived non-mammaliaform probainognathians suggests the new insertion for the *M*. *iliopsoas* occurred in basal prozostrodontians, before the origin of Mammaliaformes. This muscle is a protractor of the hindlimb in many tetrapods, including amphibians, reptiles, and mammals. The new origin and insertion of the *M*. *iliopsoas* seen in *B*. *quadrangularis* indicates an important shift of the movement generated. Indeed, the origin and insertion of this muscle are approximately at the same horizontal level in basal synapsids such as ‘pelycosaurs’. With this disposition, the muscle protracted the femur in a horizontal arc, instead of parasagittally. In *B*. *quadrangularis*, with its new area of origin and insertion, the *M*. *iliopsoas* was protracting the femur in a nearly parasagittal plane, as in living therians.

In basal synapsids and non-mammaliaform cynodonts, the *M*. *obturator externus* originated from the lateral surface of the pubis and the ischial plate, as seen in living reptiles and therians [41, 52]. In ‘pelycosaurs’ and basal non-mammaliaform cynodonts, this muscle inserted in the intertrochanteric fossa and on the apex of the lesser trochanter, as this trochanter is a crest located near the fossa and is posteriorly projected [13–14, 110]. In ‘pelycosaurs’, this muscle was probably able to retract the femur, but its main function was adduction, to elevate the body from the ground during locomotion, as seen in living reptiles [41, 52]. In mammals, the *M*. *obturator externus* is restricted to the intertrochanteric fossa because the apex of the lesser trochanter is medially located and used for the insertion of the *M*. *iliopsoas*. The morphology and the position of the lesser trochanter suggest this was also the case in *B*. *quadrangularis* and other prozostrodontians (e.g., *Prozostrodon*, *Therioherpeton*, *Irajatherium*, tritylodontids). Moreover, the morphology of the intertrochanteric fossa of *B*. *quadrangularis* is more similar to that of therians, being narrow instead of wide and circular as in other non-mammaliaform cynodonts. With the femur oriented in a nearly parasagittal plane, the adductor musculature plays a less important postural role to elevate the body from the ground and the *M*. *obturator externus* can be restricted to the intertrochanteric fossa.

### Lifestyle of *Brasilodon quadrangularis*. Fossorial adaptations

Burrow complexes containing *Galesaurus*, *Thrinaxodon* and *Langbergia* in outcrops of the Lower-Middle Triassic of South Africa suggest that fossorial activities were a common behavior in these basal non-mammaliaform cynodonts [134–136]. Moreover, complex tetrapod burrows discovered in several Lower Triassic to Jurassic continental sequences of South America, Africa and Antarctica are considered as being produced by non-mammaliaform cynodonts [137–140]. The thickened bone in the femur of the *Andescynodon* was interpreted as suggestion of a fossorial lifestyle [141]. A fossorial lifestyle for *Irajatherium*, *Riograndia*, and *Kayentatherium* was also suggested, based on scapular and humeral morphology [15–16, 21].

The humerus of *B*. *quadrangularis* lacks the prominent processes in some fossorial mammals (e.g., talpids; [94]) but several humeral features are indicative of powerful retractor muscles. The prominent teres major tuberosity indicates a massive *M*. *teres major* and this feature is seen in fossorial rodents which need great retraction capabilities to burrow [142]. Both the deltopectoral crest and the teres crest are reaching mid-shaft in *B*. *quadrangularis*, resulting in an increase of the lever arm of the *M*. *pectoralis* and *M*. *latissimus dorsi*. These muscles assisted the *M*. *teres major* to retract the humerus to the scapula. However, the ulnar olecranon process of *B*. *quadrangularis* is shorter than in *Kayentatherium* and *Haldanodon*, falling out the range of proportion (from 20% to 75%) determined by Hildebrand [123] for 27 extant genera of fossorial mammals. Moreover, the distal end of the humerus of *B*. *quadrangularis* is more gracile and narrower than the distal humeral end of *Irajatherium*, *Riograndia*, and *Kayentatherium*. In these three taxa, the distal width represents more than 50% of the humeral length (Table 2) and is associated with large muscles for borrowing [21]. This does not imply lack of burrowing capabilities for *B*. *quadrangularis*, but this cynodont had probably a more generalized lifestyle than *Irajatherium*, *Riograndia* and *Kayentatherium*.

### Scansorial adaptations

The hemispherical head of the humerus and femur indicates a higher range of rotational movements of the shoulder and the hip compare to other non-mammaliaform cynodonts, suggesting scansorial adaptations for *B*. *quadrangularis*. A more hemispherical humeral head is commonly seen in scansorial or arboreal didelphids [143] and primates [144], rather than terrestrial ones.

A reduced ulnar olecranon beak is observed in arboreal didelphids, whereas terrestrial ones have prominent olecranon beak to contain movement of flexion/extension in a strictly parasagittal plane [145]. High mobility of the elbow is also suggested by the subspherical capitulum and the nearly circular head of the radius. The oval-shaped head of the radius is a feature that can be found in more terrestrial or scansorial mammals whereas arboreal mammals have a circular shape [146]. Although the distal humeral end is narrower than in other derived non-mammaliaform probainognathians, the entepicondyle is as developed as in these taxa, representing one third of the distal end. Medially extensive entepicondyle is associated with well-developed wrist and digit flexors. The ectepicondylar (or supinator) crest is well-developed in *B*. *quadrangularis* and extends more proximally along the shaft than in *Riograndia* or *Kayentatherium*. This crest provides a large area of origin for the *M*. *brachioradialis*, which is a flexor of the antebrachium. This muscle is particularly active when the hand is in a semi-prone position and during flexion against resistance, giving it an active role during climbing [145]. It may also act as a supinator of the lower arm to bring the hands into semi-prone position so that the palmar side faces toward the medially lying arboreal substrate [62, 145]. In didelphid marsupials and tree shrew species, the entepicondyle and supinator crest are more developed in species with better climbing capability than the terrestrial species (e.g., [145, 147]).

The morphology of their shoulder, elbow and hip indicate this animal having a greater range of mobility than in other non-mammaliaform probainognathians. However, the scansorial features of *B*. *quadrangularis* shared with mammals are not necessarily correlated with arboreal habits because even dwelling on the ground, the small-sized mammals need significant climbing locomotor adaptations to overcome the irregular substrate.

### Other postcranial elements referred to *Brasilodon quadrangularis*

**Fig 12:**
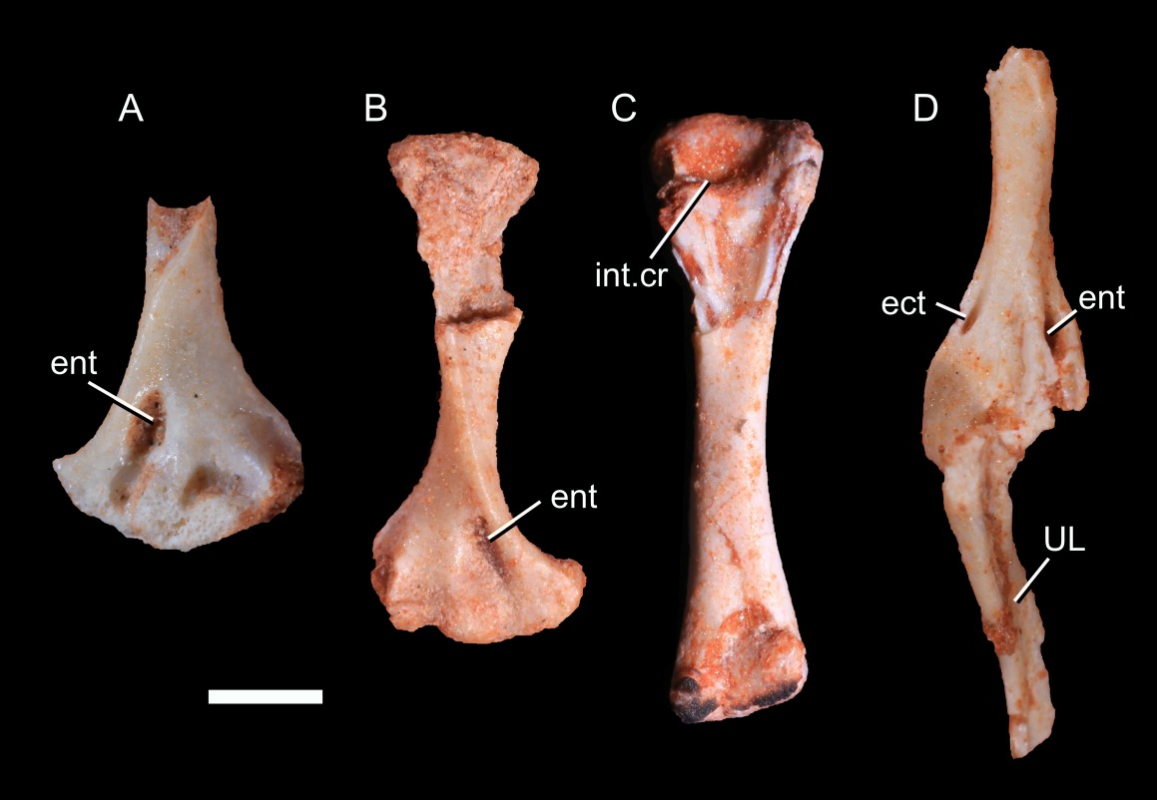
Materials previously cataloged as indeterminate brasilodontids. **A**, specimen UFRGS-PV-0600-T; **B**, specimen UFRGS-PV-1361; **C**, specimen UFRGS-PV-0922-T; **D**, specimen UFRGS-PV-1042-T, representing a new morphotype of probainognathian. **Abbreviations: ect**, ectepicondylar foramen; **ent**, entepicondylar foramen; **int.cr**, intertrochanteric crest; **UL**, ulna. Scale bar = 2 mm.

Postcranial remains referred to *Brasilodon* (or “brasilodontids”) but not associated with skull and jaw elements in the UFRGS collection include: UFRGS-PV-0778-T, UFRGS-PV-0853-T, UFRGS-PV-0922-T, UFRGS-PV-1042-T, UFRGS-PV-1361-T (Fig 12).

The specimens UFRGS-PV-0778-T and UFRGS-PV-0853-T include poorly preserved humeri and small fragments of indeterminate bones. They both are referred to *Brasilodon*, due to the lack of an ectepicondylar foramen. The specimens UFRGS-PV-0600-T and UFRGS-PV-1361-T include respectively the distal end of a left and a right humerus. Both humeri are smaller in size than that of UFRGS-PV-1043-T, and their entepicondyles are less prominent. These slight differences can be due to ontogenetic stage, considering the small size of UFRGS-PV-0922-T, UFRGS-PV-0600-T and UFRGS-PV-1361-T, in comparison to the specimen UFRGS-PV-1043-T of *B*. *quadrangularis*.

The specimen UFRGS-PV-1042-T includes the distal end of a small left humerus articulated with the proximal part of the left ulna. The distal end differs considerably from that of *B*. *quadrangularis* in having an ectepicondylar foramen and a well-marked olecranon fossa. This humerus also differs significantly from those of *Irajatherium hernandezi* [15] and *Riograndia guaibensis* [21], which have stout humeri, with possibly semi-fossorial adaptations. This material may represent a new species of basal probainognathian from the *Riograndia* AZ.

The specimen UFRGS-PV-0922-T includes a small and isolated right femur, which is similar to the femur of UFRGS-PV-1043-T, with a narrow intertrochanteric fossa, the presence of a short femoral neck and salient intertrochanteric crest. The femur of UFRGS-PV-0922-T differs from that of UFRGS-PV-1043-T, in having a less distinct greater trochanter and a narrower distal end. However, these differences can be due to ontogenetic variation, considering their difference in size.

## Conclusion

The postcranium of *B*. *quadrangularis* differs from other non-mammaliaform cynodonts and is similar to early mammaliaforms and extant therians (e.g., hemispherical humeral and femoral head, distinct greater tubercle of the humerus, circular acetabulum, salient intertrochanteric crest of the femur). The morphology of the ulnar condyle of the humerus and olecranon process of the ulna suggests more abilities for extension and flexion of the elbow, a necessary component for a parasagittal locomotion. However, the humeral torsion, the length of the deltopectoral crest, the large bicipital groove and the well-developed lesser tubercle, indicate that the forelimb of *B*. *quadrangularis* was hold in a semi-sprawling position, with well-developed adductor muscles to maintain the body off the ground. The short femoral neck and the strong medial projection of the femoral head indicate the femur was held in a more erect posture than in basal non-mammaliaform cynodonts. The anterodorsally projected iliac blade with reduced postacetabular process, enlarged obturator foramen, reduction of the anterior part of the pubis, prominent and distinctive greater trochanter, medially located lesser trochanter, narrow intertrochanteric fossa represent a further continuation of trends that indicates a basically mammalian pattern of pelvic musculature, able to swing the femur in a nearly parasagittal plane.

**Fig 13:**
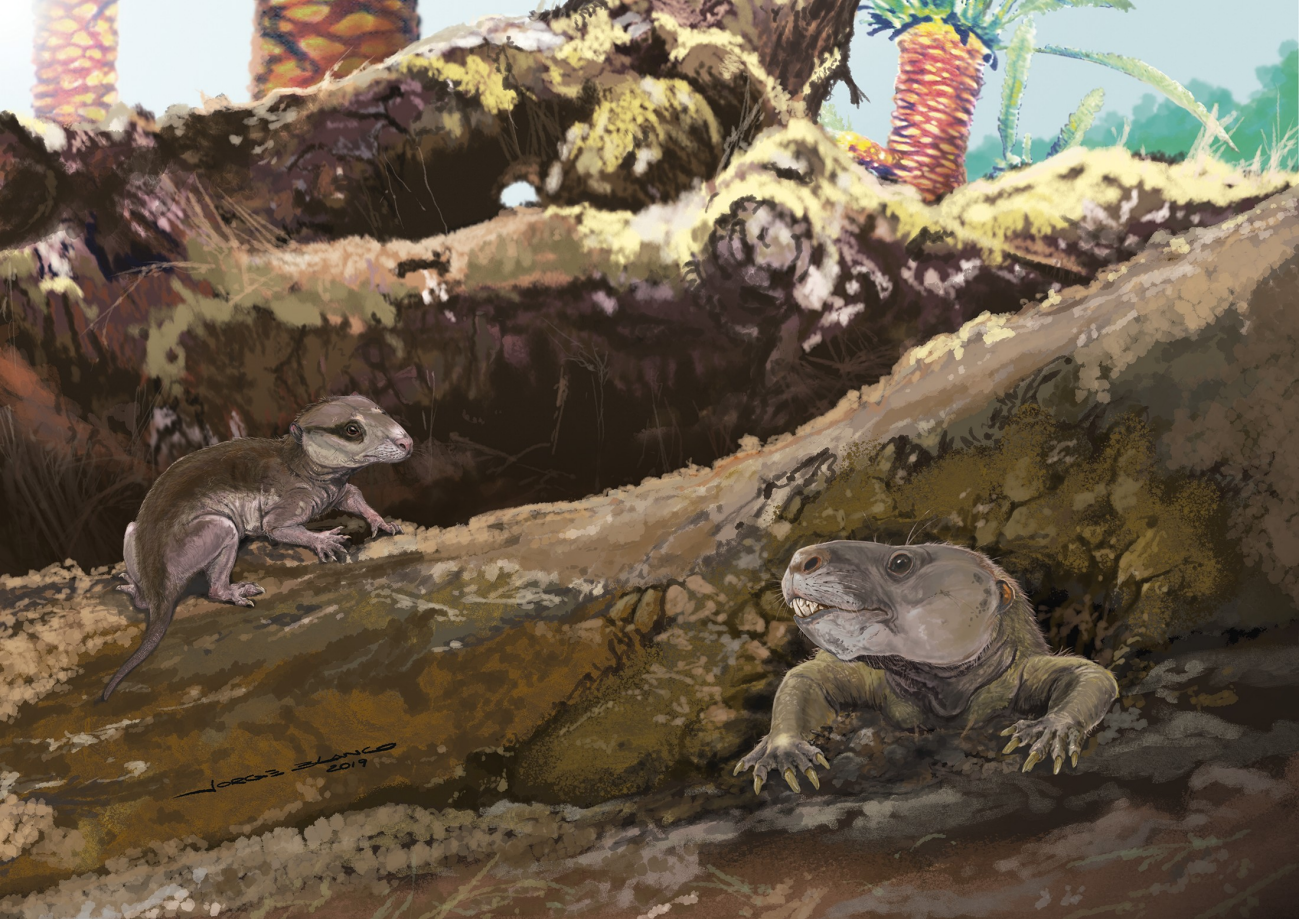
Paleoartistic reconstruction of *Brasilodon quadrangularis* (left) and *Riograndia guaibensis* (right), two abundant probainognathian cynodonts from the *Riograndia* AZ of the Candelária Sequence, Santa Maria Supersequence (Brazil), which exhibits different morphologies in skull, dentition and locomotor apparatus. Made by Jorge Blanco.

Although fossorial or semifossorial habits appear to have been common in derived non-mammaliaform probainognathians (i.e., *Riograndia*, *Irajatherium* and *Kayentatherium*), the postcranial study of *B*. *quadrangularis* shows more generalized adaptations and highlights that distinctive ecological strategies (Fig 13) were developed among small-sized non-mammaliaform cynodonts.
